# Supplementary material for: Neurostructural subgroup in 4291 individuals with schizophrenia identified using the subtype and stage inference algorithm
Source: Nat Commun. 2024 Jul 17;15:5996. doi: 10.1038/s41467-024-50267-3 (PMC11252381; doi:10.1038/s41467-024-50267-3)
Supplement: Supplementary file 1 — Supplementary Information [file 41467_2024_50267_MOESM1_ESM.pdf]

Supplementary Material

Neurostructural subgroup in 4291 individuals with schizophrenia identified using the Subtype and Stage Inference algorithm

Jiang et al.

This supplementary material includes as follows:

Assumptions about schizophrenia for SuStaIn.
Supplementary Table 1. Sample demographics by participating cohort.
Supplementary Table 2. Dataset-specific information.
Supplementary Table 3. Demographic and clinical characteristics of participants in the first-episode subsample and medication-naive subsample.
Supplementary Table 4. Demographic and clinical characteristics of participants in six cohorts by their locations.
Supplementary Table 5. Morphological z-scores of all brain regions and inter-subtype comparisons.
Supplementary Table 6. Difference of volume of striatum between the subtype1 and subtype2 in a medication-naïve subsample.
Supplementary Table 7. AAL3 brain regions.
Supplementary Table 8. Description of SuStaIn features.
Supplementary Figure 1. Pathophysiological progression trajectories in validation dataset.
Supplementary Figure 2. Pathophysiological progression trajectories in first-episode population and medication-naïve population.
Supplementary Figure 3. Comparisons of morphological z-score between the two subtypes.
Supplementary Figure 4. Hippocampus subregional morphological z-score for the two subtypes.
Supplementary Figure 5. Amygdala subregional morphological z-score for the two subtypes.
Supplementary Figure 6. Generalization of SuStaIn subtyping and staging to unseen cohorts.
Supplementary Figure 7. Significant association between regional gray matter volume change and illness duration in 2,333 patients with schizophrenia.
Supplementary Figure 8. The consistency of individual classification in both original model and replicated model.
Acknowledgements per dataset
References

Assumptions about schizophrenia for SuStaIn

In our modelling approach, we assume that disease progression is a linear deviation from the normality of a patient's brain profile. In this context, it is important to distinguish anatomical progression from clinical progression. Concerning positive symptoms, progressive deviation from normality does not occur in schizophrenia. Most patients show a degree of symptomatic amelioration over a long time, despite recurrent periods of exacerbation [1]. Concerning negative symptoms, despite some early improvement, a cumulative pattern with pronounced deficits is seen in several patients receiving psychiatric care [2]. Concerning the grey matter volume, when cross-sectional studies across various illness stages are considered, a pattern of spatial expansion of structural changes, as well as an increase in magnitude (effect size) of localized changes are noted. A subtle increase in grey matter is also a feature of schizophrenia, and this increase may appear in the early stages [3, 4]. To date, such subtle increases have not been shown to 'reverse' the early grey matter reduction to the point of return to normality [5]. Thus, the assumption that a lack of atrophy reflects the earliest stages of illness, while progressively later stages show more deviation from normality is thus reasonable based on the extant literature on structural changes in schizophrenia.

We also assume that tonic changes rather than event-related changes (relapse) occur in schizophrenia. There are not many empirical data to directly address this question, but the amount of time spent in a symptomatic state, rather than the number of relapses, has a direct relationship to grey matter reduction in the presence of psychosis [6, 7]. As residual symptom burden is the norm rather than an exception in the schizophrenia [8], tonic changes in grey matter volume are a reasonable expectation, though the rate of this change may vary with the stage of illness.

When interpreting our modelling approach, it is worth noting that the presence of pre-onset neurodevelopmental brain abnormalities per se does not preclude a specific post-onset pattern of disease progression. The model we apply is agnostic as to the origins of the structural deficits seen at the onset; in other words, the model considers baseline deficits (e.g., insular volume reduction in subtype1) to have resulted from multiple processes (including neurodevelopmental deviation) but allows for the same pattern of deficits (i.e., insular volume loss) to occur later in the illness via putative degenerative or compensatory pathways in the subtype2. As a result, the assumptions required to interpret our model to accommodate pre-existing, putatively developmental, structural deficits.

Similar to dementias, no brain region is known to consistently display higher grey matter volume at later stages compared to earlier stages of schizophrenia to date (in adults) (for example, see Koutsouleris et al. [9]). When we consider individualized centile scores for grey matter volume in the context of normative age-related trends [10]: schizophrenia closely follows Alzheimer's disease, with volume reduction in schizophrenia being more pronounced than in mild cognitive impairment (MCI). Thus, while we apply a modelling approach (SuStaIn) that is mostly used for the neurodegenerative condition, the similarities in the spatiotemporal patterns of structural changes between dementias and schizophrenia allow us to translate the model to a non-degenerative condition.

Supplementary Table 1. Sample demographics by participating cohort.

Cohort	Location	N		Removed subjects by quality control	Mean Age (SZ)	Mean Age (HC)	F/M (SZ)	F/M (HC)	Mean Duration Of Illness (years)	PANSS Positive Subscale	PANSS Negative Subscale	PANSS General Subscale	PANSS Total Score	SAPSTOT	SANSTOT
	Country/Region	Case	Control												
IGP	Australia	68	71	0	41.7±11.1	36.0±11.0	28/40	32/39	18.8±9.5	13.8±5.7	14.5±6.1	27.2±9.4	55.5±18.4	14.7±11.3	20.5±12.9
ESO	Czech Republic	40	40	0	29.5±7.0	29.1±6.5	20/20	20/20	0.6±0.8	14.2±5.6	16.1±5.1	33.6±9.5	63.8±17.4	-	-
FIDMAG	Spain	158	123	1	39.5±11.8	37.5±10.1	35/123	69/54	15.4±11.2	16.8±5.7	22.6±6.8	36.8±9.3	76.2±18.2	-	37.2±13.0
FOR2107-MR	German	90	563	4	38.2±11.9	36.3±13.0	43/47	347/216	16.0±10.7	-	-	-	-	12.9±13.2	13.1±10.3
FOR2017-MS	German	37	386	3	36.8±11.0	31.3±12.2	18/19	255/131	15.3±10.1	-	-	-	-	15.2±13.3	14.2±14.4
IMH	Singapore	141	0	0	33.2±9.0	-	46/95	-	6.5±7.2	10.7±3.9	9.0±3.0	20.5±3.8	40.2±8.5	-	-
JBUN	Korea	116	0	2	34.8±12.9	-	60/56	-	5.7±7.6	14.0±6.4	13.2±7.0	27.0±8.6	54.2±19.1	-	-
OLIN	USA	73	264	2	41.1±13.1	39.9±14.0	29/44	124/140	16.9±12.8	-	-	-	-	-	-
Osaka	Japan	310	1180	8	35.4±12.8	33.6±14.1	143/167	581/599	11.4±9.4	19.6±5.9	20.7±5.8	44.4±11.0	84.7±21.0	-	-
PENS	USA	20	16	0	47.4±9.5	45.9±10.1	7/13	10/6	21.7±9.4	-	-	-	-	22.0±14.7	26.7±13.7
PHCP	USA	41	38	0	42.2±11.6	38.4±13.7	11/30	17/21	17.2±10.4	-	-	-	-	21.1±14	35.6±14.1
RomeSL	Italy	162	114	8	39.5±11.3	37.4±11.6	53/109	43/71	15.0±10.7	20.9±6.4	21±7.4	45.0±11.5	86.9±20.2	31.7±19.5	28.9±16.0
SoCAT	Turkey	113	79	5	36.6±8.4	37.3±9.0	37/76	31/48	14.9±6.8	11.6±5.1	19.7±7.5	30.2±6.3	61.3±13.9	-	44.2±21.4
SWIFT	Switzerland	24	13	0	34.2±10.9	29.3±4.0	7/17	8/5	9.5±7.8	16.4±5.4	12.8±5.0	28.8±6.9	58.0±12.0	-	-
UCISZ	USA	26	29	0	42.9±10.8	41.7±12.3	5/21	7/22	17.3±10.2	15.5±4.2	16.2±6.0	28.2±6.9	59.9±12.2	14.4±9.2	24.0±14.5
UNINA	Italy	49	55	0	37.5±9.7	42.4±15.7	15/34	26/29	14.7±8.2	19.2±5.3	22.3±5.9	44.0±9.8	85.5±17.2	-	-
Zurich	Switzerland	60	28	0	30.5±8.5	32.5±9.3	15/45	10/18	8.4±7.3	10.7±2.7	14.5±5.9	23.4±4.8	48.7±10.4	-	24.9±15.7
COBRE*	USA	79	86	9	37.8±13.8	38.7±11.9	15/64	23/63	15.6±12.6	15.2±5.0	15.2±5.4	29.2±9.1	59.6±15.6	-	-
TOPSY	Canada	58	31	4	23.3±4.6	21.5±3.5	12/46	12/19	0.6±1.1	-	-	-	-	-	-
Voices	Australia	38	47	4	43.4±10.4	31.5±12.0	21/17	25/22	1.8±0.9	17.8±5.6	13.8±4.8	34.2±10.6	65.9±18.2	18.9±14.0	17.7±12.9
OSLO_TOP	Norway	389	769	5	31.2±9.3	33.5±8.8	159/230	344/425	-	-	-	-	-	-	-
CN-Shanghai1*	China	361	267	7	24.3±8.0	24.0±4.9	167/194	144/123	0.9±1.4	20.2±5.7	15.4±7.4	38.1±8.1	73.7±15.7	-	-
CN-Shanghai2	China	159	133	1	24.9±7.0	23.7±5.9	84/75	67/66	1.4±2.0	23.7±5.3	18.1±6.8	41.4±7.2	83.2±13.7	-	-
CN-Shanghai3	China	42	23	0	29.9±7.4	31.2±5.9	23/19	12/11	6.6±5.7	19.9±3.1	18.4±6.4	33.1±4.9	71.4±9.0	-	-
CN-Harbin	China	49	0	0	26.7±8.1	-	22/27	-	2.4±3.2	20.2±4.5	22.4±6.4	40.4±7.4	83.0±14.5	-	-
CN-Chengdu*	China	101	127	3	40.2±11.8	38.1±14.9	31/70	42/85	15.5±10.3	13.2±5.8	21.0±6.3	28.2±5.7	62.3±13.1	-	-
CN-Taibei*	China	158	254	8	43.9±11.0	38.0±12.0	93/65	136/118	15.7±10.1	9.6±3.2	10.0±5.5	20.7±4.4	40.3±10.7	-	-
CN-Zhengzhou*	China	194	59	1	23.2±8.6	26.5±5.5	97/97	32/27	-	22.3±5.1	23.1±6.3	44.1±7.9	89.5±15.0	-	-

CN-Beijing1	China	130	0	9	21.3±4.2	-	75/55	-	-	22.4±5.0	20.9±7.2	38.8±7.0	82.1±15.1	-	-
CN-Beijing2	China	217	721	3	26.3±6.6	24.8±4.2	85/132	365/356	-	-	-	-	-	-	-
CN-Changsha	China	120	92	2	23.4±5.5	23.5±5.2	39/81	51/41	-	15.3±5.8	18.3±8.8	31.9±10.6	65.4±22.0	-	-
CN-Xian	China	39	51	0	23.9±8.6	21.1±4.0	17/22	18/33	1.2±1.8	22.9±4.9	21.4±6.4	44.8±8.8	89.1±14.6	-	-
HCP-EP	USA	54	39	1	21.9±3.0	25.4±4.4	15/39	14/25	-	11.7±3.9	16.0±5.6	24.9±4.5	52.7±10.1	-	-
JP-SRPBS	Japan	139	887	10	38.3±10.8	36.5±15.5	58/81	382/505	14.4±9.6	14.6±5.3	17.0±6.4	31.4±9.4	62.9±18.9	-	-
fBIRN*	USA	42	65	6	38.1±11.8	38.3±11.2	14/28	29/36	-	-	-	-	-	-	-
MCIC	USA	109	95	1	33.8±11.2	32.9±12.2	26/83	30/65	11.1±10.9	-	-	-	-	-	-
NMorphCH*	USA	44	43	0	32.5±6.9	31.5±8.4	14/30	22/21	-	-	-	-	-	-	-
NUSDAST*	USA	123	127	1	33.8±12.5	32.6±13.8	42/81	62/65	-	-	-	-	-	-	-
DS000030	USA	50	121	1	36.5±8.9	31.6±8.8	12/38	56/65	-	-	-	-	-	-	-
DS000115*	USA	22	18	0	24.2±3.8	20.7±4.8	6/16	8/10	-	-	-	-	-	-	-
DS004302	Spain	46	24	0	42.5±10.7	39.5±14.3	10/36	7/17	-	-	-	-	-	-	-
Total	-	4291	7078	109	32.5±11.9	33.0±12.7	1709/2582	3461/3617	10.5±10.4	17.2±6.8	17.5±7.6	34.8±11.6	69.5±22.4	22.0±17.5	27.1±18.0

Note: * some of these cohorts overlap with a previous paper (https://doi.org/10.1038/s44220-023-00024-0)

Supplementary Table 2. Dataset-specific information.

Cohort	Diagnosis measurement	Exclusion/inclusion criteria	Scanner manufacturer and type	Imaging protocols
IGP	ICD-10	All participants were fluent English speakers and aged 18-65 years old. No history of an organic brain disorder, brain injury accompanied by > 24 h of amnesia, mental retardation defined as an IQ < 70, movement disorder, current substance dependence, or electro-convulsive therapy in the preceding 6 months. The control participants additionally had no personal history of psychotic disorder or family history of psychotic disorder in their first-degree biological relatives.	1.5T Siemens Avanto	High-resolution T1-weighted structural magnetic resonance imaging (sMRI) brain scans (MPRAGE) were acquired using an optimized magnetization prepared rapid acquisition gradient echo on 1.5 T Siemens Avanto scanners (Siemens, Erlangen, Germany) across five Australian research sites. Image parameters were set to 176 slices of 1mm thickness, no gap with field-of-view 250 x 250 mm2, repetition time 1980 ms, echo time 4.3 ms, data acquisition matrix 256 x 256, with a flip matrix of 15°, resulting in a voxel size of 0.98×0.98×1.0mm3.
ESO	ICD-10 (F20.x, F23, F25)	Early or first-episode psychosis, Czech language as a mother tongue, 18-60 years old. Neurocognitive disorders (organic mental disorder), mental disorders caused by addiction, mental retardation (IQ<80), severe neurological disorder, head injury, hypertension, cerebrovascular disease, epilepsy, migraine, endocrine disorders.	3T Siemens Tim Trio	MP-RAGE 3D, 1mm thickness, acquisition matrix 256 x 256, TR=2300ms, TE=4.63ms,TI=900ms
FIDMAG	DSM-IV criteria based on interview and review of clinical history	Patients had a diagnosis of schizophrenia. All participants were in the 18-65 age range. Controls were excluded if they reported a history of mental illness and/or treatment with psychotropic medication. Patients were excluded if have had a history of brain trauma or neurological disease or had shown alcohol/ substance abuse within 12 months before participation	1.5T GE Signa	180 axial slices; 1mm slice thickness, no gap, matrix size 512x512; 0.5x0.5x1mm3 voxel resolution; TE 4ms, TR 2000ms, flip angle 15.
FOR2107-MR	DSM-IV-TR using SCID-I semi-structured interview	All participatnts were in the 18-65 age range; patients were diagnosed of schizophrenia, schizoaffective disorder or any schizophreniform disorder. Exclusion criteria all: any history of neurological (head trauma or unconsciousness) and medical condition (severe somatic disorders), IQ<80; Exclusion criteria controls: any current or former psychiatric disorder; Exclusion criteria patients: Current benzodiazepine treatment (wash out of at least three half-lives before study participation).	3T Siemens Magnetom TrioTim Syngo	MPRAGE imaging sequence. 1 acquisition. Flip angle: 9 degrees. TE: 2.26 ms. TR: 1900 ms. TI: 900 ms. Acceleration factor: 2Field of view: 256. Image dimensions: 256x256x176 voxels. Voxel size: 1x1x1 mm.
FOR2017-MS	DSM-IV-TR using SCID-I semi-structured interview	All participatnts were in the 18-65 age range; patients were diagnosed of schizophrenia, schizoaffective disorder or any schizophreniform disorder. Exclusion criteria all: any history of neurological (head trauma or unconsciousness) and medical condition (severe somatic disorders), IQ<80; Exclusion criteria controls: any current or former psychiatric disorder; Exclusion criteria patients: Current benzodiazepine treatment (wash out of at least three half-lives before study participation).	3T Siemens PRISMA	MPRAGE imaging sequence. 1 acquisition. Flip angle: 8 degrees. TE: 2.28 ms. TR: 2130 ms. TI: 900 ms. Acceleration factor: 2. Field of view: 256. Image dimensions: 256x256x192 voxels. Voxel size: 1.0x1.0x1.0mm

IMH	SCID DSM-IV Axis I Disorders	(1) Diagnosis of SZ (Patients), (2) Age: 21-65, (3) English speaking, (4) Provision of informed written consent. History of significant head injury; significant Neurological diseases (such as epilepsy, cerebrovascular accident) or Medical Illnesses; significant DSM IV alcohol or substance use or dependence; contraindications to MRI (e.g. pacemaker, orbital foreign body, recent surgery/procedure with metallic devices/implants deployed); pregnant women; claustrophobia.	3T Philips Achieva	T1 scans: 180 axial slices of 0.9mm thickness with no gap, FOV = 230x230 mm2, matrix 256x204, voxel size =0.89x0.89x0.9 mm3, TR=7.2 s, TE=3.3 ms, FA=8°.
JBUN	DSM-V	A diagnosis of schizophrenia: all patients were between 18 – 71 years old. Patients with an (a) alcohol or substance use disorder; (b) intellectual disability (IQ ≤70); (c) current or past neurological disease, serious medical illness, or pregnancy; and (d) claustrophobia were excluded.	3T Siemens MAGNETOM Verio syngo	T1-weighted, 3-dimensional Magnetization Prepared Rapid Gradient Echo (TR = 1900ms; TE=2.45ms; FOV=250mm; FA=9°; voxel size=1x1x1mm3; Slice thickness=1mm).
OLIN	SCID	SCID-NP	3T Siemens Allegra	T1-weighted, 3D magnetization prepared rapid gradient-echo (MPRAGE) sequence (TR/TE/TI=2200/4.13/766 ms, flip angle=13°, voxel size [isotropic]=0.8mm, image size=240 x 320 x 208 voxels), with axial slices parallel to the AC-PC line.
Osaka	SCID-P; DSM-IV	SCID-NP	1.5T GE Signa Excite; 3T GE Signa HDxt	3D-IR-FSPGR, TR/TE/TI=12.6/4.2/400ms, flip angle=15°, 256x256x124 matrix, FOV=240x240mm, slice thickness=1.4mm, Nex=1, No Asset, QD Head coil; 3D-IR-FSPGR, TR/TE/TI=7.2/2.9/400ms, flip angle=11°, 256x256x172 matrix, FOV=240x240mm, slice thickness=1.0mm, Nex=1, No Asset, 8ch Brain coil.
PENS	DSM-IV	Same as PHCP study: Adults aged 18–65, including people with major mental illness (schizophrenia, schizoaffective disorder, bipolar I disorder with psychotic features), their first-degree biological relatives, and unrelated healthy controls.	3 Tesla Siemens Prisma scanner using a 32 channel head coil	Structural MRI using a 10-min T1-weighted MPRAGE sequence (TE = 2.12 ms, TR = 2,400 ms, flip angle = 8, resolution = 256)
PHCP	DSM-IV	All participants spoke English as their primary language and did not have: a legal guardian (or otherwise lack capacity to provide informed consent), alcohol/drug abuse in the past month or alcohol/drug dependence in the last 6 months, a diagnosed Learning Disability or estimated IQ lower than 70 (if either condition was diagnosed based on testing by a trained professional or the latter by research staff), a current or past central nervous system disease (including: seizures, epilepsy, encephalitis, MS, Parkinson's, stroke), history of head injury with skull fracture or loss of consciousness greater than 30 min, history of electro-convulsive therapy (ECT) in the last year, tardive dyskinesia (as evidenced by medical record), obstructed or compromised vision (e.g., lazy eye that is uncorrected or was corrected after age 17 / strabismus / cross eyes / permanent eye injury / abnormality in visual field / cataract), hearing problems (e.g., cannot hear without hearing aid / severe tinnitus), or a condition likely making it impossible to perform tasks (e.g., paralysis, severe arthritis).	Siemens 3 T Prisma scanner with a Siemens 32 channel head coil	A multi-echo T1w MPRAGE sequence and a variable-flip-angle, turbo-spin-echo T2w scan with volumetric navigators to aid real-time motion correction and selective reacquisition were acquired with scanning protocol identical to that of the Lifespan Human Connectome Project (see Harms et al., 2018). As in the Lifespan HCP (Harms et al., 2018), up to 30 k-space lines for the T1w scan and up to 25 k -space lines for the T2w scan were allotted for reacquisition. T1w MPRAGE multi-echo (300 × 320 matrix, FOV=240 × 256mm, resolution=0.8mm, flip angle=8, TE=1.81, 3.6, 5.39, 7.18 ms, TR=2500ms, slices/orientation=208 sag, AF=2, time=8min:22sec

		Additionally, patients were between the ages of 18 and 65 years old with a diagnosis of schizophrenia, schizoaffective disorder, or bipolar I disorder with a history of psychotic symptomatology (i.e., delusions or hallucinations) with no indication that symptoms were caused by substance use or a general medical condition. While patients were screened and excluded for current substance use issues, a history of such issues as well as current/lifetime comorbidities of any kind were permitted for enrollment in the study in order to have a sample representative of patients with psychosis in the general population while simultaneously limiting nuisance effects. Finally, as this was a family study, patients and relatives were not adopted.<br><br>Biological relatives were 18–69 years old with a first-degree biological relative with schizophrenia, schizoaffective disorder, or bipolar I disorder with psychosis, and living within one day's drive or planning to visit the vicinity of the University of Minnesota. Because relatives included parents of PwP, we expanded the age range of the relatives group to accommodate recruitment efforts. Relatives were enrolled regardless of psychopathology. Approximately half (45.95%) of the first-degree biological relatives in the study carried their own mental health diagnosis (e.g., major depression). Occasionally, relatives with substance dependence in partial remission (3 participants, 3.9%), current substance dependence (1 participant, 1.3%), or psychotic psychopathology (1 participant, 1.3%) were included in the study.<br><br>Controls were aged 18–65 and had no history of schizophrenia, schizoaffective disorder, or bipolar I disorder with psychotic features, or other psychotic symptoms or history of major depressive disorder. Additionally, controls had no first-degree biological relative with a history of psychiatric hospitalization for a psychotic or affective disorder.		
RomeSL	SCID DSM-IV Axis I Disorders (SCID-I) and SCID DSM-IV Axis II Personality Disorders (SCID-II)	Inclusion criteria were (i) age between 18 and 65 years; (ii) at least five years of education; and (iii) suitability for MRI scanning. Exclusion criteria were (i) history of alcohol or drug abuse in the two years before the assessment; (ii) lifetime drug dependence; (iii) traumatic head injury with loss of consciousness; (iv) past or present major medical illness or neurological disorders; (v) any (for HC) or additional (for patients) psychiatric disorder or mental retardation; (vi) dementia or cognitive deterioration according to DSM-IV-TR criteria, and Mini-Mental State Examination (MMSE) score < 25, consistent with normative data in the Italian population; (vii) not able and willing to give written informed consent.	Siemens 3T Allegra	3D MPRAGE: TE/TR = 2.4/7.92 ms, flip angle=15°, voxel size 1×1×1 mm.

SoCAT	DSM-V; DSM-IV	Inclusion: (1) Aged between 19-45, (2) had been followed up for at least one year with schizophrenia diagnosis according to the medical records, (3) had confirmed diagnosis of schizophrenia and no other axis I diagnosis after evaluation with SCID, (4) were clinically stable over the past three months (no change in symptoms requiring interventions such as medication change or hospitalization) and had cognitive abilities enough to read and understand the informed consent form. Exclusion: (1) an unstable medical disease (e.g., diabetes mellitus, hypertension, etc.), (2) a history of head trauma with loss of consciousness, (3) contraindications to MRI (e.g. pacemaker, orbital foreign body, recent surgery/procedure with metallic devices/implants deployed); pregnant women; claustrophobia.	3T Siemens Magnetom Verio; 1.5T Siemens SymphonyVision	Sagittal T1-weighted 3D magnetization-prepared rapid gradient echo acquisition (MPRAGE) sequence (TE = 2.21 msec, TR = 1600 msec, TI = 900 msec, FA = 9, FOV = 256, voxel size = 0.5x0.5x1 mm, number of slices = 160, no inter-slice gap). Sagittal T1-weighted 3D magnetization-prepared rapid gradient echo acquisition (MPRAGE) sequence (TE = 3.93 msec, TR = 2300 msec, TI = 1100 msec, FA = 12, FOV = 256, voxel size = 0.5x0.5x1 mm, number of slices = 160, no inter-slice gap).
SWIFT	SCID DSM-IV-TR	Age between 18-65 y/o, good German language skills that allowed them to understand the consent procedure and to undergo the clinical assessment, right-handedness according to the Edinburgh Inventory. For patients: Diagnosis with a schizophrenia spectrum disorder. Participants were excluded if they were left-handed, pregnant, showed any contraindications for MRI (e.g. metal-containing implants such as pacemaker or cochlear implants, claustrophobia), had a history of serious neurological issues, or reported current abuse of alcohol and/or psychoactive substances (apart from nicotine). Additionally, controls had no current major psychiatric DSM-IV Axis I diagnoses, as assessed with the screening questionnaire of the Structured Clinical Interview for DSM-IV Axis I Disorders.	3T Siemens Verio	MPRAGE: 160 sagittal slices, 1mm slice thickness, 256x256 matrix size, 1x1x1 mm3 voxel size. TR = 2.3ms, TE = 2.98ms, TI = 900ms.
UCISZ	SCID DSM-IV-TR criteria	All subjects diagnosed with schizophrenia were clinically stable outpatients whose antipsychotic medications and doses had not changed within the last two months. Schizophrenia and healthy volunteers with a history of major medical illness, drug dependence in the last five years (except for nicotine), current substance abuse disorder, or MRI contraindications, were excluded. Individuals with schizophrenia who had significant tardive dyskinesia and healthy volunteers with a current or past history of major neurological or psychiatric illness or with a first-degree relative with an Axis-I psychotic disorder diagnosis were also excluded.	3T Philips Achieva	T1TFE:200 sagittal slices, 320x274 matrix size, .75mm isotropic, TR = 11ms, TE=4.562ms, flip angle = 18°
UNINA	DSM-5	Schizophrenia:(1) Diagnosis of SZ (Patients); (2) Age: 18-60; (3) Provision of informed written consent; (4) Disease duration >2 years; (5) no medication switch or dose changes in the last 6 months (i.e., >10% baseline dose); (6) no evidence of current or recent (3 months) worsening of psychotic symptoms; absence of: (7) macroscopic brain structural anomalies; (8) major systemic disorder (such as cardiovascular, endocrine, metabolic disorders); (9) other psychiatric disorder (including addiction disorder, substance use disorder, or frequent substance use in the 6 months preceding the recruitment); (10) moderate or severe neurological disorder; (11) intellectual disability; (12) pregnancy or lactation; (13) enrollment in any sort of experimental clinical trial within 3 months	3T Siemens Tim Trio	3D T1-weighted Magnetization Prepared Rapid Acquisition Gradient Echo sequence (MPRAGE; TR=1900 ms; TE=3.4 ms; TI=900 ms; Flip Angle=9°; resolution=1x1x1 mm3; 160 axial slices).

		from recruitment.<br>Controls:(1) No psychiatric diagnosis; (2) Age: 18-60; (3) Provision of informed written consent; 4) Absence of: macroscopic brain structural anomalies; major systemic disorder (such as cardiovascular, endocrine, metabolic disorders); moderate or severe neurological disorder; intellectual disability; pregnancy or lactation		
Zurich	MINI DSM IV	A diagnosis of schizophrenia. We excluded patients with any other DSM-IV Axis I disorder (in particular, current substance use disorder and major depressive disorder), those medicated with lorazepam at a dose higher than 1 mg, those with florid psychotic symptoms (i.e., any positive subscale item scores higher than 4 on the PANSS scale and those with extrapyramidal side effects (i.e., a total score higher than 2 on the MSAS). Healthy controls were screened for any neuropsychiatric disorders using the structured Mini-International Neuropsychiatric Interview to ensure that they had no previous or present psychiatric illness. Both patients and healthy controls were required to have a normal physical and neurologic status and no history of major head injury or neurologic disorder.	3T Philips	3D T1-weighted images were acquired with an ultra-fast gradient echo T1-weighted sequence (TR=8.4ms, TE=3.8ms, flip angle=8°) in 160 sagittal plan slices (1mm slice thickness, no slice gap) of 240×240mm2 resulting in 1x1x1mm3voxels.
COBRE	SCID DSM-IV Axis I Disorders	All participants were in the 18-65 age range and had a diagnosis of schizophrenia. Healthy individuals were included if they did not have a personal or family history of psychiatric disorders. History of neurological disorder, history of mental retardation, history of severe head trauma with more than 5 minutes loss of consciousness, history of substance abuse or dependence within the last 12 months and MRI contraindications.	3T Siemens TIM Trio	T1-weighted images were acquired with a 5-echo multi-echo MPRAGE sequence [TE (echo times) = 1.64, 3.5, 5.36, 7.22, 9.08 ms, TR (repetition time) = 2.53 s, TI (inversion time) = 1.2 s, 7° flip angle, number of excitations (NEX) = 1, slice thickness = 1 mm, FOV (field of view) = 256 mm, resolution = 256x256].
TOPSY	DSM-V	Inclusion criteria for FEP: individuals experiencing FEP, with lifetime antipsychotic treatment less than 14 days. Exclusion criteria for FEP: meeting criteria for a mood disorder (bipolar or major depressive) with psychotic features, or possible drug-induced psychosis. Healthy control (HC) participants were free from personal history of mental illness or family history of psychotic disorders, matched based on age, sex, and parental education. Exclusion criteria for both FEP and HC: substance use disorder in the past year based on DSM-5 criteria, history of major head injury, significant medical illness, or contraindications to MRI.	Siemens MAGNETOM 7.0T MRI Plus (Siemens, Erlangen, Germany)	Anatomical images were acquired using a sagittal 3D MP2RAGE1 sequence with TE=2.83 ms, TR=6000 ms, TI=800ms/2700 ms, flip angle=4/5 degrees, matrix=320x320x208, iPAT=3, partial Fourier=6/8, voxel size=0.8x0.8x0.8, and a 3D SA2RAGE2, with TE=0.81 ms, TR=2400 ms, TI=45/1800 ms, flip angle=4o/5o, matrix=320x320x208, iPAT=3, partial Fourier=6/8, voxel size=0.8mmx0.8mmx0.8mm.
Voices	MINI	Inclusion: diagnosis of schizophrenia or schizoaffective disorder. Age: 18-65, fluent in English, Able to provide informed consent.<br>Exclusion: History of significant head injury or neurological disease affecting cognition (such as epilepsy). Current DSM IV alcohol or substance use or dependence; contraindications to MRI (e.g. pacemaker, orbital foreign body, recent surgery/procedure with metallic devices/implants deployed); currently pregnant	3T Siemens Tim Trio	Images were optimized using a magnetization-prepared rapid acquisition gradient echo (MP-RAGE) sequence, and consisted of 176 sagittal slices/brain of 1 mm thickness without gap; field of view = 256 × 256 mm2; repetition time/echo time =1900/2.52 ms; data matrix size =256 × 256; voxel dimensions =1.0 × 1.0 ×1.0 mm3. All scans were conducted at a single site.

OSLO_TOP	The Structured Clinical Interview for DSM-IV axis 1 disorders (SCID-IV).	Healthy controls were randomly drawn from the national population registry in the same geographical area as the patients, and invited by letter to participate. They were screened prior to participation. Absence of current or previous history of a psychiatric disorder was determined by self-report. Current symptomatology was screened for on the day of inclusion using the Prime MD and alcohol and drug use were screened for using AUDIT/DUDIT.<br>The exclusion criteria for healthy controls were: - Age outside of the range 18-65 years. - Current or previous psychiatric disorder. - History of severe mental illness in a first-degree relative. - Any alcohol or drug abuse or dependence	1.5T Siemens Magnetom Sonata	Two sagittal T1-weighted magnetization prepared rapid gradient echo (MPRAGE) volumes were acquired with the Siemens tfl3d1_ns pulse sequence (TE = 3.93 ms, TR = 2730 ms, TI = 1000 ms, flip angle = 7°; FOV = 24 cm, voxel size= 1.33 x 0.94 x 1 mm3, number of partitions = 160).
CN-Shanghai1	DSM-IV	All the subjects were Mandarin-speaking Han Chinese individuals from Shanghai metropolitan area. The FES patients were identified according to DSM-IV criteria by qualified psychiatrists using all available clinical information including a diagnostic interview of patients and their family, clinical case notes, and clinician's observations. All healthy subjects were assessed in accordance with DSM-IV criteria as being free of schizophrenia and other axis I disorders, and none had neurological diseases, head trauma, or substance abuse. All participants were right-handed; had no history of substance abuse or suicidal ideation; and had no MRI contraindications.	GE Sigma 3T	GE Sigma 3.0 T MR (GE Medical Systems, Milwaukee, Wisconsin). TR = 7.8 ms; TE = 3.65 ms; flip angle = 7°; matrix = 256×256; voxel size = 1×1×1 mm.
CN-Shanghai2	DSM-IV	The exclusion criteria were as follows: (1) brain trauma, substance-related disorders, major medical or neurologic disorders; (2) other mental disorders meeting DSM-IV criteria; (3) drug or alcohol abuse; (4) pregnancy, breastfeeding or other unstable clinical state including aggressive behaviour; (5) history of electroconvulsive therapy or transcranial magnetic stimulation within six months; and (6) other contraindications to MRI scanning. To eliminate potential familial effects, healthy subjects whose first- or second-degree relatives had a history of mental disorders were also excluded.	3T Siemens MR B17	A 3-Tesla MRI scanner (Siemens MR B17) was used to acquire data in the Shanghai Mental Health Centre. High-spatial-resolution T1-weighted images were collected by a magnetization-prepared rapid acquisition gradient echo (MPRAGE) sequence. The main parameters included repetition time/echo time, 2530/2.56 msec; flip angle, 7°; field of view, 256 × 256 mm2; matrix size, 256 × 256; section thickness, 1 mm (no gap); and voxel size, 1×1×1mm3.
CN-Shanghai3	DSM-IV-TR	Inpatients with schizophrenia were recruited from the Shanghai Mental Health Center (SMHC). All the patients were recruited from October 2013 to January 2015. All patients met criteria for schizophrenia or schizoaffective disorder based on the Structured Clinical Interview for Diagnostic and Statistical Manual of Mental Disorders (DSM-IVTR) as conducted by senior psychiatrists. The severity of psychotic symptoms was assessed by the Positive and Negative Syndrome Scale (PANSS). All patients had a total PANSS score of 60 or more and had not received ECT during the past six months. All health controls did not have a lifetime psychiatric disorder or family history of psychosis in their first-degree relatives. Participants were excluded if they had brain injuries, organic mental disorders, neurologic abnormalities, other serious physical illnesses, dementia, substance abuse or dependence, or contraindications to MRI.	3-T Siemens Magnetom Verio Syngo MR B17	High-resolution T1-weighted images were acquired using a 3-T Siemens Magnetom Verio Syngo MR B17 scanner. High-resolution T1-weighted images were collected with a magnetization-prepared rapid acquisition gradient echo (MPRAGE) sequence (TR = 2530 ms; TE = 2.56 ms; flip angle = 7°; inversion time = 1100 ms; FOV = 256 mm × 256 mm; matrix = 256 × 256; slice thickness = 1 mm; 224 slices; and voxel size = 1.0 × 1.0 × 1.0 mm).

CN-Harbin	DCM-IV SCID	Patient recruitment criterion was based on the Diagnostic and Statistical Manual of Mental Disorders, Fourth Edition and Structured Clinical Interview for DSM (SCID), which includes: (1) Age range between 14-45 years old; (2) Intelligence quotient (IQ)>69; (3) The first onset and no systematic antipsychotic treatment before admission; (4) Psychotic symptom assessment scale (positive and negative symptom scale score) is greater than or equal to 60; clinical overall impression CGI severity is greater than or equal to 4 points; (5) Exclude other Axis I mental disorders except schizophrenia, and have no DSM-5 drug or alcohol dependence within the past three months. Exclusion criteria for patients included the following: (1) Sensory-motor disturbances (hearing impairment, blindness), neurological disorders (brain injury, epilepsy), or other medical conditions (2) claustrophobic and unable to undergo MRI scans; (3) patients with repetitive transcranial magnetic stimulation or MRI Contraindications to scanning, such as those with metal implants in the body; (4) suicide attempters, pregnant or breastfeeding women.	3.0 Tesla GE Discovery MR750	All scans were acquired on the same 3.0 Tesla GE Discovery MR750 scanner equipped with a 32-channel head coil.
CN-Chengdu	DSM-IV	Patients with SZ were recruited from the Clinical Hospital of Chengdu Brain Science Institute. Each patient was diagnosed based on the Diagnostic and Statistical Manual of Mental Disorders, Fourth Edition (DSM-IV). Subjects with a history of brain injuries, substance-related disorders, major medical or neurological disorder were excluded. To exclude the potential effect of similar genetic backgrounds, the history of psychiatric disorder in a first- or second-degree relative was an additional exclusion criterion for healthy controls.	3-Tesla GE DISCOVERY MR 750	A 3-Tesla MRI scanner (GE DISCOVERY MR 750, USA) was used to collect imaging data in the University of Electronic Science and Technology of China. High-resolution T1-weighted images were acquired using a three dimensional fast spoiled gradient echo (T1-3D FSPGR) sequence. The main parameters include: TR = 6.008 ms; TE = 1.984 ms; flip angle (FA) =90°; field of view (FOV) = 25.6 cm × 25.6 cm; matrix size = 256 × 256; slice thickness = 1 mm (no gap).
CN-Taibei	DSM-IV	Patients and matched healthy controls were recruited from the Veteran General Hospital in Taipei, Taiwan. All participants were diagnosed according to the Diagnostic and Statistical Manual of Mental Disorder-IV criteria for schizophrenia, and each participant's history of medical disease, psychiatric illness, and medication use was evaluated by interview and medical charts carefully. Any participants with the following conditions were excluded: (1) a comorbid substance-related disorder, (2) presence of neurobiological disorders, such as dementia, head injury, stroke, or Parkinson’s disease; (3) presence of hypertension, diabetes, hyperlipidemia or coronary heart disease; (4) severe medical illness, such as malignancy, heart failure, or renal failure; (4) presence of ferromagnetic foreign bodies or implants that were anywhere in the body.	3T Siemens	Siemens MAGNETOM Tim Trio 3.0T MRI Scanner (Siemens Healthcare, Erlangen, Germany) with a 12- channel head coil, using 3-dimensional magnetization prepared rapid gradient-echo sequence. TR: 2530 ms, TE: 3.5 ms, TI: 1,100 ms, FoV: 256 mm, flip angle: 7 degree, 192 sagittal slices, voxel size = 1.0 mm3 cubic, no gap.
CN-Zhengzhou	DSM-IV	All patients were identified according to the Diagnosis and Statistic Manual of Mental Disorders, fourth edition (DSM-IV) criteria for schizophrenia by qualified psychiatrists using all available clinical information including a diagnostic interview, clinical case notes, and clinician’s observations. The severity of positive and negative symptoms was assessed by trained and experienced psychiatrists using PANSS scoring. Individuals were excluded from the study if they were diagnosed with	GE MR750 3T	GE MR750 3T MRI scanner with standard quadrature head coil. TR/TE= 8.2 / 3.2 ms, FOV= 256×256 mm 2, Matrix= 256×256, slice thickness= 1.0 mm, gap= 0mm, 188 contiguous sagittal slices；flip angle = 12°.

		schizoaffective disorder, mood disorders, other cognitive disorders, epilepsy, had severe physical diseases, alcohol/drug dependence, or been treated with electroconvulsive therapy. Healthy subjects were assessed in accordance with DSM-IV criteria as being free of schizophrenia and other Axis I disorder, and none had neurological diseases, head trauma, substance abuse, suicidal ideation, and MRI contraindications.		
CN-Beijing1	DSM-IV	The inclusion criteria were: (1) both in- and out-patients, (2) both genders, (3) diagnosis of schizophrenia established with Structured Clinical Interview for the DSM-IV Axis I Disorder, patient edition, (4) aged between 18 and 45 years, (5) onset at age ≥ 15 years, (6) first episode of schizophrenia, (7) no previous psychiatric treatment ('drug-naïve' status), and (8) ability to understand the contents of interview and provide written informed consent.<br>The exclusion criteria were: (1) a history or diagnosis of major medical conditions, (2) a history of alcohol and / or drug abuse or dependence, and (3) contra-indication to olanzapine, aripiprazole, or risperidone.	Siemens Trio Trim 3.0 T	Main parameters: TR: 9.8 ms, TE: 3.8 ms, TI: 450 ms, FoV: 512 x 512, flip angle: 13, slice thickness=1.2 mm.
CN-Beijing2	DSM-IV	A consensus diagnosis of schizophrenia was made by two experienced senior psychiatrists according to the Diagnosis and Statistic Manual of Mental Disorders, fourth edition (DSM-IV) criteria for schizophrenia or schizophreniform disorder using the Structured Clinical Interview for DSM-IV-TR Axis I Disorders, Patient Edition (SCID-I/P). All subjects initially recruited with schizophreniform disorder were finally diagnosed with schizophrenia after being followed up for at least six months. Individuals were excluded from the study if they were diagnosed with schizoaffective disorder, mood disorders, delusional disorder, brief psychotic disorder, psychosis associated with substance use or medical conditions, learning disability, pervasive developmental disorder, delirium, dementia, amnesia or other cognitive disorders; had severe, unstable physical diseases (such as diabetes, thyroid diseases, hypertension and cardiac diseases), a well-documented history of epilepsy, a DSM-IV diagnosis of alcohol or drug dependence; had been treated with electroconvulsive therapy within the last six months; were pregnant or breastfeeding; had previously attempted suicide; or had experienced symptoms of severe excitement and agitation within one week before MRI scanning. Healthy control individuals were recruited from each hospital and screened using SCID-I, Non-Patient Edition (SCID-I/NP). Individuals with any history of mental disorders or first- or second-degree relatives with any history of mental disorders were excluded.	Siemens Trio Trim 3.0 T	T1-weighted images were collected with matrix size of 256×256, resolution of 1×1 mm2, and slice thickness of 1 mm.
CN-Changsha	DSM-IV SCID-P	The inclusion/exclusion criteria of patients: (1) diagnosis of SCZ confirmed using the Structured Clinical Interview for DSM-IV-patient version (SCID-P) 1; (2) Han Chinese ethnicity and right-handed; (3) minimum 9 years of school education (4) good general physical health with no known	Philips Gyroscan Achieva 3T	3-D structural MRI images (T1-weighted) were acquired from the sagittal plane using spoiled gradient echo (SPGR) pulse sequence on a Philips Gyroscan Achieva 3T MRI scanner, scanning parameter: TR=12 ms,

		endocrine, metabolic or inflammatory disorders; (5) no known neurological disorder; (6) no substance dependence in the last year; (7) benzodiazepine treatment, if any, stopped more than 24 h prior to scanning and (8) no contraindications for MRI.<br>The inclusion/exclusion criteria of healthy subjects in two samples were identical to those of the patient group except that they did not meet the DSM-IV diagnostic criteria of any mental disorders screened using the SCID non-patient version. We also ensured that for healthy subjects, no first-degree relatives had a history of any psychiatric disorders.		TE=4.2 ms, flip angle=15°, 172 slices, matrix size=256×256, and the field of view (FOV)=24×24 cm2.
CN-Xian	DSM-IV, DSM-V	Inclusion criteria were the following: (1) diagnosis of schizophrenia using the Structured Clinical Interview for the Diagnostic and Statistical Manual of Mental Disorders, Fourth Edition (SCID-IV), (2) patients were taking stable doses of antipsychotic medication for at least 8 weeks before the study; (3) no history of significant head trauma or neurological disorders and no focal brain lesions by T1- or T2-weighted MRI; (4) no alcohol or drug abuse; and (5) patient has no recent aggression or other forms of behavioral dysfunction.	GE Discovery MR750 3.0 T	High-resolution T1-weighted MRI was acquired using a GE Discovery MR750 3.0 T scanner located in the Department of Radiology, and all subjects underwent T2WI scans to rule out organic diseases. The scanning parameters were repetition time 8.2 ms, echo time 3.2 ms, flip angle 12°, field of view 256 × 256 mm, matrix 256 × 256, slice thickness 1 mm, and sagittal slices 196.
HCP-EP	DSM-V	Available at https://www.humanconnectome.org/study/human-connectome-project-for-early-psychosis		
JP-SRPBS		Available at https://bicr-resource.atr.jp/srpbsfc/		
fBIRN	SCID DSM-IV-TR	All subjects diagnosed with schizophrenia were clinically stable outpatients whose antipsychotic medications and doses had not changed within the last two months. Schizophrenia and healthy volunteers with a history of major medical illness, drug dependence in the last five years (except for nicotine), current substance abuse disorder, or MRI contraindications, were excluded. Individuals with schizophrenia who had significant tardive dyskinesia and healthy volunteers with a current or past history of major neurological or psychiatric illness or with a first-degree relative with a DSM IV Axis-I psychotic disorder diagnosis were also excluded.	3T Siemens Tim Trio; 3T GE	High-resolution structural imaging scans were acquired on six 3T Siemens Tim® Trio System and one 3T General Electric Discovery MR750 scanner. MP-RAGE scan parameters for the Siemens scanner were: scan plane=sagittal, TR/TE/TI=2300/2.94/1100ms, GRAPPA acceleration factor=2, flip angle=9°, resolution=256×256x160, FOV=220mm2, voxel size=0.86x0.86x1.2mm, and NEX=1. IR-SPGR scan parameters for the General Electric scanner were: scan plane=sagittal, TR/TE/TI=5.95/1.99/450ms, ASSET acceleration factor=2, a flip angle=12°, resolution=256×256x166, FOV=220mm2, voxel size=0.86x0.86x1.2mm, and NEX=1. All scans covered the entire brain.
MCIC	SCID DSM-IV (SCID-NP for controls) or CASH were used to diagnose primary and co-morbid psychiatric disorders in	All subjects were between the ages of 18 and 60 and spoke English as their native language. To be included in the schizophrenia cohort, patients had to meet diagnostic criteria for schizophrenia, schizoaffective disorder, or schizophreniform disorder. Concerted effort was made to recruit patients early in the course of their illness and especially those who were antipsychotic drug naïve. The healthy control subjects with no current or past history of psychiatric illness including substance abuse or dependence were matched within site to the patient cohort for age, sex, and parental education. Control subjects who had not been diagnosed with any psychiatric disorders, but had been medicated with antidepressants, anti-anxiety medication or medication for sleep disturbance	1.5, 3T Siemens and GE	T1 scans: TR = 2530 ms for 3 T, TR = 12 ms for 1.5 T; TE = 3.79 ms for 3 T, TE = 4.76 ms for 1.5 T; FA = 7 for 3 T, FA = 20 for 1.5 T; TI = 1100 for 3 T; Bandwidth = 181 for 3 T, Bandwidth = 110 for 1.5 T; 0.625×0.625 mm voxel size; slice thickness 1.5 mm; FOV 256×256×128 cm matrix; FOV = 16 cm (could be increased to 18 cm when needed for full brain coverage).

	controls and patients	were included in the study provided that the duration of their medication did not exceed 2 months of lifetime use and no medication was used within the 6 months preceding the baseline MRI scan. Control subjects who met criteria for current or past history of substance abuse or dependence were excluded from the study. Patients, however, were not excluded from the study unless criteria were met for current (i.e., within the past month) abuse or dependence (except for 6 patients who were found to meet criteria for current abuse after the study data was collected). Both patients and controls were excluded if they had (1) an IQ less than 70 based on a standardized IQ test, (2) history of a head injury resulting in prolonged loss of consciousness, neurosurgical procedure, neurological disease, history of skull fracture, severe or disabling medical conditions, or (3) a contraindication for MRI scanning such as pregnancy, metal in body or head including implanted pacemaker, medication pump, vagal stimulator, deep brain stimulator, implanted TENS unit, or ventriculo-peritoneal shunt		
NMorphCH	This is a longitudinal study examining the clinical, cognitive and neuroimaging (MRI) data from schizophrenia and control subjects at baseline and after two years. The study was conducted at Northwestern University. Neuroimaging data includes T1, T2, DTI, resting-state fMRI and n-back task fMRI. More details are provide at http://www.schizconnect.org/documentation		3T	MPRAGE:voxel size =1x1x1.6 mm3; Matrix size 256x256; TR=3.15ms; TE=20ms; Flip ange=8.0
NUSDAST	The Northwestern University Schizophrenia Data and Software Tool (NUSDAST) is a repository of schizophrenia neuroimaging data collected from over 450 individuals with schizophrenia, healthy controls and their respective siblings, most with 2-year longitudinal follow-up. More details are provided at: Wang L, Kogan A, Cobia D, et al. Northwestern University schizophrenia data and software tool (NUSDAST). Frontiers in neuroinformatics, 2013, 7: 25.		Siemens 1.5 T Vision scanner	3D MPRAGE (TR = 9.7 ms, TE = 4 ms, flip = 10°, ACQ = 1, 256 × 256 matrix, 1 × l mm in-plane resolution, 128 slices, slice thickness 1.25 mm)
DS000030	DSM-IV	For both healthy and patient groups, participants were men or women ages 21–50 years; NIH racial/ethnic category either White, not Hispanic or Latino; or Hispanic or Latino, of any racial group; primary language either English or Spanish; completed at least 8 years of formal education; no significant medical illness; adequate cooperation to complete assessments; visual acuity 20/60 or better; and urinalysis negative for drugs of abuse (Cocaine; Methamphetamine; Morphine; THC; and Benzodiazepines).<br>Participants in the healthy group were excluded if they had lifetime diagnoses of Schizophrenia or Other Psychotic Disorder, Bipolar I or II Disorder, or Substance Abuse or Dependence (not counting caffeine or nicotine); or current Major Depressive Disorder; suicidality; Anxiety Disorder (Obsessive Compulsive Disorder, Panic Disorder, Generalized Anxiety Disorder, Post-Traumatic Stress Disorder), Attention Deficit Hyperactivity Disorder (ADHD). ADHD criteria were assessed using Adult ADHD Interview; healthy participants were screened for sub-threshold ADHD, defined as 4 or more ADHD inattentive or hyperactive/impulsive symptoms in either childhood and adulthood; in addition, they could not have had medication treatment for ADHD within the prior 12	Siemens Trio (2 Imaging Sites)	A T1-weighted high-resolution anatomical scan (MPRAGE) were collected with the following parameter: slice thickness = 1mm, 176 slices, TR=1.9s, TE=2.26ms, matrix=256 x 256, FOV=250mm.

		months. Each of the patient groups (Schizophrenia, Bipolar Disorder, and ADHD) excluded anyone with one of these other diagnoses; stable medications were permitted for the patients. For MRI studies we excluded participants who were left handed, who believed they might be pregnant, or had other contraindications to scanning (e.g., claustrophobia, metal in body, body too large to fit in scanner).<br>More details are provided at https://www.nature.com/articles/sdata2016110		
DS000115	DSM-IV	All subjects were diagnosed on the basis of a consensus between a research psychiatrist who conducted a semi-structured interview and a trained research assistant who used the Structured Clinical Interview for DSM-IV Axis I Disorders. Participants were excluded if they: (a) met DSM-IV criteria for substance dependence or severe/moderate abuse during the prior 6 months; (b) had a clinically unstable or severe medical disorder; (c) had a history of head injury with documented neurological sequelae or loss of consciousness; or (d) met DSM-IV criteria for mental retardation. The individuals with schizophrenia were all outpatients, and were stabilized on antipsychotic medication for at least 2 weeks. Controls were required to have no lifetime history of Axis I psychotic or mood disorders and no first-degree relatives with a psychotic disorder.	3T Siemens Trio	T1 structural image was acquired using a sagittal MP-RAGE 3D sequence (TR = 2400 ms, TE = 3.16 ms, flip = 8°; voxel size = 1 mm × 1 mm × 1 mm).
DS004302	DSM-V	Patients meeting DSM-5 criteria for schizophrenia were initially recruited from four psychiatric hospitals in Barcelona. Diagnoses were made using the Structured Clinical Interview for DSM Disorders (SCID). Patients were excluded if they (a) were younger than 18 or older than 65, (b) had a history of brain trauma or neurological disease or (c) had shown alcohol/substance abuse/dependence within 12 months prior to participation. Social use of alcohol was permitted, as was non-habitual use of cannabis. Electroconvulsive therapy in the past 6 months was also an exclusion criterion. All participants were right-handed and were taking antipsychotic medication. Healthy controls met the same exclusion criteria as the patients, and they were also interviewed using the SCID to exclude current and past psychiatric disorders. They were questioned and excluded if they reported a history of treatment with psychotropic medication beyond non-habitual use of night sedation. Controls were also excluded if they reported a history of major psychiatric disorder in a first-degree relative.	3T Philips Ingenia scanner	Images were acquired with a 3T Philips Ingenia scanner (Philips Medical Systems, Best, The Netherlands). High-resolution anatomical volume with an FFE (Fast Field Echo) sequence for anatomical reference and inspection (TR = 9.90ms; TE = 4.60ms; Flip angle = 8°; voxel size = 1 × 1mm; slice thickness = 1mm; slice number = 180; FOV = 240mm).

Supplementary Table 3. Demographic and clinical characteristics of participants in the first-episode subsample and medication-naive subsample.

	First-episode cohort (n=1122)		Medication-naïve cohort (n=718)	
	n	mean(SD)	n	mean(SD)
Sex (Female/Male)	513/609	-	353/365	-
Age (years)	1122	25.4(8.6)	718	23.7(7.8)
Illness duration (years)	710	0.78(0.67)	185	1.3(2.2)
PANSS Positive scale (P1-P7)	926	19.5(6.4)	605	21.1(5.5)
PANSS Negative scale (N1-N7)	926	17.0(7.3)	605	18.5(7.8)
PANSS General scale (G1-G16)	926	37.6(10.3)	605	39.6(8.1)
PANSS Total score	926	74.1(20.2)	605	79.2(16.6)
PANSS excitement dimension (P4, P7, G44, G14)	597	8.8(3.4)	533	8.7(3.3)
PANSS depression/anxiety dimension (G1, G2, G3, G6, G15)	597	11.3(3.8)	533	11.0(3.6)
PANSS cognitive dimension (P2, N5, G5, G10, G11)	597	10.1(3.7)	533	10.0(3.7)

Supplementary Table 4. Demographic and clinical characteristics of participants in six cohorts by their locations.

	East Asian ancestry cohort (*n*=2235)		European ancestry cohort (*n*=1987)		China cohort (*n*=1542)		Japan cohort (*n*=438)		Europe cohort (*n*=1044)		North American cohort (*n*=729)	
	n	mean(SD)	*n*	mean(SD)	*n*	mean(SD)	*n*	mean(SD)	*n*	mean(SD)	*n*	mean(SD)
Sex (Female/Male)	1017/1218	-	666/1321	-	718/824	-	195/243	-	374/670	-	207/522	-
Age (years)	2235	29.8(11.5)	1987	35.3(11.6)	1542	27.4(10.5)	438	36.0(12.1)	1044	35.1(11.1)	729	34.4(12.4)
Illness duration (years)	1228	7.7(9.1)	1105	13.5(11.0)	587	5.7(8.6)	386	11.9(9.4)	580	13.3(10.7)	359	13.6(12.0)
PANSS Positive scale (P1-P7)	1847	17.8(6.9)	804	15.8(6.3)	1180	18.9(6.9)	413	18.2(6.1)	448	17.4(6.5)	152	14.0(4.9)
PANSS Negative scale (N1-N7)	1847	17.1(7.8)	804	18.3(7.2)	1180	17.6(8.0)	413	19.6(6.2)	448	19.8(7.3)	152	15.7(5.6)
PANSS General scale (G1-G16)	1847	35.2(11.6)	804	34.0(11.6)	1180	35.9(10.4)	413	40.6(12.1)	448	37.9(12.3)	152	27.7(7.8)
PANSS Total score	1847	70.1(22.9)	804	68.0(21.2)	1180	72.4(21.0)	413	78.4(22.6)	448	75.1(22.2)	152	57.4(13.9)
PANSS excitement dimension (P4, P7, G44, G14)	770	8.7(3.4)	552	7.5(3.4)	691	9.0(3.4)	79	6.1(2.1)	406	8.2(3.5)	79	4.9(1.4)
PANSS depression/anxiety dimension (G1, G2, G3, G6, G15)	770	11.0(3.5)	552	11.6(4.9)	691	11.2(3.5)	79	9.7(3.2)	406	12.3(4.8)	79	8.2(3.0)
PANSS cognitive dimension (P2, N5, G5, G10, G11)	770	10.2(3.7)	552	11.1(4.3)	691	10.3(3.7)	79	9.0(3.2)	406	11.7(4.6)	79	9.7(2.8)
Cohorts included	IMH; JBUN; Osaka; CN-Shanghai1; CN-Shanghai2; CN-Shanghai3; CN-Harbin; CN-Chengdu; CN-Taibei; CN-Zhengzhou; CN-Beijing1; CN-Beijing2; CN-Changsha; CN-Xian; JP-SRPBS		IGP; ESO; FIDMAG; FOR2107-MR; FOR2017-MS; OLIN; PENS; PHCP; RomeSL; SoCAT; SWIFT; UCISZ; UNINA; Zurich; COBRE; TOPSY; Voices; OSLO_TOP; HCP-EP; fBIRN; MCIC; NMorphCH; NUSDAST; DS000030; DS000115; DS004302		CN-Shanghai1; CN-Shanghai2; CN-Shanghai3; CN-Harbin; CN-Chengdu; CN-Taibei; CN-Zhengzhou; CN-Beijing1; CN-Beijing2; CN-Changsha; CN-Xian		Osaka; JP-SRPBS		ESO; FIDMAG; FOR2107-MR; FOR2017-MS; RomeSL; SWIFT; UNINA; Zurich; OSLO_TOP; DS004302		OLIN; PENS; PHCP; UCISZ; COBRE; TOPSY; HCP-EP; fBIRN; MCIC; NMorphCH; NUSDAST; DS000030; DS000115	

Supplementary Table 5. Morphological z-scores of all brain regions and inter-subtype comparisons.

Number	Region	Morphological measure (Freesurfer)	Subtype1 (*n*=1,131)		Subtype2 (*n*=709)		Subtype1 vs. Subtype2			
			Mean	SD	Mean	SD	Cohen's d	*t*	Two-sided *p*	FWE
1	Left_Lateral_Ventricle	Subcortical volume	-0.186	0.992	-0.459	1.355	0.230	4.971	7.297E-07	*
2	Left_Thalamus	Subcortical volume	0.196	1.123	0.449	1.090	-0.228	-4.745	2.251E-06	*
3	Left_Caudate	Subcortical volume	-0.264	1.034	0.279	1.073	-0.516	-10.815	0.000E+00	*
4	Left_Putamen	Subcortical volume	-0.204	1.023	0.213	0.990	-0.415	-8.623	0.000E+00	*
5	Left_Pallidum	Subcortical volume	-0.273	0.987	0.083	0.931	-0.371	-7.699	2.232E-14	*
6	Left_Hippocampus	Subcortical volume	0.169	0.988	0.586	0.821	-0.460	-9.393	0.000E+00	*
7	Left_Amygdala	Subcortical volume	0.096	0.968	0.399	0.897	-0.325	-6.724	2.358E-11	*
8	Left_Accumbens_area	Subcortical volume	0.080	1.040	0.383	1.051	-0.290	-6.056	1.691E-09	*
9	Right_Lateral_Ventricle	Subcortical volume	-0.206	1.143	-0.429	1.317	0.181	3.835	1.297E-04	*
10	Right_Thalamus	Subcortical volume	0.224	1.132	0.457	1.147	-0.205	-4.279	1.970E-05	*
11	Right_Caudate	Subcortical volume	-0.268	1.061	0.240	1.109	-0.468	-9.820	0.000E+00	*
12	Right_Putamen	Subcortical volume	-0.211	0.983	0.181	1.040	-0.388	-8.144	6.661E-16	*
13	Right_Pallidum	Subcortical volume	-0.291	0.985	0.051	0.987	-0.347	-7.245	6.323E-13	*
14	Right_Hippocampus	Subcortical volume	0.152	1.041	0.594	0.918	-0.449	-9.246	0.000E+00	*
15	Right_Amygdala	Subcortical volume	0.005	1.004	0.370	0.920	-0.379	-7.825	8.438E-15	*
16	Right_Accumbens_area	Subcortical volume	0.083	0.937	0.377	0.989	-0.305	-6.402	1.941E-10	*
17	Left_Lateral_nucleus	Amygdala segmentation volume	0.029	0.919	0.374	0.822	-0.395	-8.144	6.661E-16	*
18	Left_Basal_nucleus	Amygdala segmentation volume	0.116	0.952	0.451	0.829	-0.375	-7.713	1.998E-14	*
19	Left_Accessory_Basal_nucleus	Amygdala segmentation volume	0.144	0.964	0.488	0.861	-0.376	-7.753	1.477E-14	*
20	Left_Anterior_amygdaloid_area_AAA	Amygdala segmentation volume	0.142	0.985	0.352	0.887	-0.224	-4.620	4.110E-06	*
21	Left_Central_nucleus	Amygdala segmentation volume	-0.002	0.932	0.179	0.919	-0.195	-4.065	5.014E-05	*
22	Left_Medial_nucleus	Amygdala segmentation volume	-0.018	0.943	0.138	0.954	-0.165	-3.437	6.009E-04	
23	Left_Cortical_nucleus	Amygdala segmentation volume	0.029	0.981	0.322	0.963	-0.301	-6.274	4.382E-10	*
24	Left_Corticoamygdaloid_transitio	Amygdala segmentation volume	0.198	0.962	0.536	0.843	-0.374	-7.685	2.465E-14	*
25	Left_Paralaminar_nucleus	Amygdala segmentation volume	0.047	0.979	0.388	0.848	-0.372	-7.644	3.375E-14	*
26	Right_Lateral_nucleus	Amygdala segmentation volume	0.007	0.964	0.402	0.898	-0.425	-8.792	0.000E+00	*
27	Right_Basal_nucleus	Amygdala segmentation volume	0.074	1.020	0.491	0.938	-0.426	-8.812	0.000E+00	*
28	Right_Accessory_Basal_nucleus	Amygdala segmentation volume	0.122	1.001	0.500	0.953	-0.387	-8.027	1.776E-15	*
29	Right_Anterior_amygdaloid_area_AAA	Amygdala segmentation volume	0.106	0.976	0.337	0.952	-0.239	-4.980	6.955E-07	*
30	Right_Central_nucleus	Amygdala segmentation volume	-0.008	0.972	0.207	0.970	-0.222	-4.629	3.931E-06	*

31	Right_Medial_nucleus	Amygdala segmentation volume	-0.047	0.989	0.146	0.998	-0.194	-4.059	5.134E-05	*
32	Right_Cortical_nucleus	Amygdala segmentation volume	0.017	1.001	0.315	1.011	-0.296	-6.192	7.311E-10	*
33	Right_Corticoamygdaloid_transitio	Amygdala segmentation volume	0.174	1.023	0.559	0.961	-0.388	-8.038	1.665E-15	*
34	Right_Paralaminar_nucleus	Amygdala segmentation volume	0.010	1.013	0.423	0.924	-0.426	-8.789	0.000E+00	*
35	Left_Hippocampal_tail	Hippocampus segmentation volume	0.149	0.989	0.442	0.973	-0.299	-6.217	6.244E-10	*
36	Left_subiculum_body	Hippocampus segmentation volume	0.050	0.979	0.403	0.900	-0.376	-7.761	1.388E-14	*
37	Left_CA1_body	Hippocampus segmentation volume	0.129	1.012	0.256	0.999	-0.126	-2.633	8.538E-03	
38	Left_subiculum_head	Hippocampus segmentation volume	0.023	0.989	0.312	0.881	-0.309	-6.361	2.517E-10	*
39	Left_hippocampal_fissure	Hippocampus segmentation volume	-0.169	0.975	0.011	0.991	-0.183	-3.829	1.332E-04	*
40	Left_presubiculum_head	Hippocampus segmentation volume	0.072	0.984	0.372	0.900	-0.319	-6.585	5.916E-11	*
41	Left_CA1_head	Hippocampus segmentation volume	0.125	1.000	0.451	0.842	-0.353	-7.226	7.249E-13	*
42	Left_presubiculum_body	Hippocampus segmentation volume	0.095	0.965	0.353	0.948	-0.269	-5.608	2.359E-08	*
43	Left_parasubiculum	Hippocampus segmentation volume	0.123	1.014	0.209	1.029	-0.084	-1.756	7.933E-02	
44	Left_molecular_layer_HP_head	Hippocampus segmentation volume	0.145	0.995	0.471	0.833	-0.355	-7.266	5.458E-13	*
45	Left_molecular_layer_HP_body	Hippocampus segmentation volume	0.198	0.980	0.513	0.892	-0.336	-6.929	5.851E-12	*
46	Left_GC_ML_DG_head	Hippocampus segmentation volume	0.189	1.022	0.446	0.859	-0.273	-5.587	2.657E-08	*
47	Left_CA3_body	Hippocampus segmentation volume	0.066	0.979	0.208	0.994	-0.144	-3.000	2.735E-03	
48	Left_GC_ML_DG_body	Hippocampus segmentation volume	0.188	0.987	0.438	0.929	-0.261	-5.399	7.551E-08	*
49	Left_CA4_head	Hippocampus segmentation volume	0.165	1.023	0.404	0.876	-0.251	-5.149	2.898E-07	*
50	Left_CA4_body	Hippocampus segmentation volume	0.182	0.967	0.425	0.913	-0.258	-5.348	9.993E-08	*
51	Left_fimbria	Hippocampus segmentation volume	0.049	1.119	0.265	1.053	-0.199	-4.122	3.918E-05	*
52	Left_CA3_head	Hippocampus segmentation volume	0.118	1.003	0.298	0.923	-0.187	-3.868	1.137E-04	*
53	Left_HATA	Hippocampus segmentation volume	0.200	0.978	0.452	0.847	-0.276	-5.658	1.771E-08	*
54	Right_Hippocampal_tail	Hippocampus segmentation volume	0.111	1.001	0.461	0.968	-0.355	-7.380	2.383E-13	*
55	Right_subiculum_body	Hippocampus segmentation volume	0.046	0.974	0.400	0.907	-0.376	-7.791	1.099E-14	*
56	Right_CA1_body	Hippocampus segmentation volume	0.064	1.037	0.231	0.982	-0.165	-3.420	6.398E-04	
57	Right_subiculum_head	Hippocampus segmentation volume	0.007	1.074	0.327	0.974	-0.312	-6.435	1.574E-10	*
58	Right_hippocampal_fissure	Hippocampus segmentation volume	-0.285	1.071	-0.081	1.050	-0.193	-4.013	6.240E-05	*
59	Right_presubiculum_head	Hippocampus segmentation volume	0.096	1.051	0.409	1.004	-0.304	-6.311	3.461E-10	*
60	Right_CA1_head	Hippocampus segmentation volume	0.105	1.088	0.466	0.948	-0.354	-7.278	4.993E-13	*
61	Right_presubiculum_body	Hippocampus segmentation volume	0.099	1.037	0.392	1.013	-0.285	-5.935	3.495E-09	*
62	Right_parasubiculum	Hippocampus segmentation volume	0.143	1.029	0.304	1.034	-0.155	-3.245	1.195E-03	
63	Right_molecular_layer_HP_head	Hippocampus segmentation volume	0.133	1.083	0.492	0.946	-0.354	-7.275	5.101E-13	*

64	Right_molecular_layer_HP_body	Hippocampus segmentation volume	0.150	1.035	0.495	0.980	-0.343	-7.106	1.707E-12	*
65	Right_GC_ML_DG_head	Hippocampus segmentation volume	0.164	1.045	0.427	0.918	-0.268	-5.507	4.160E-08	*
66	Right_CA3_body	Hippocampus segmentation volume	0.010	0.972	0.164	0.946	-0.160	-3.330	8.841E-04	
67	Right_GC_ML_DG_body	Hippocampus segmentation volume	0.134	1.041	0.405	1.016	-0.263	-5.479	4.856E-08	*
68	Right_CA4_head	Hippocampus segmentation volume	0.144	1.023	0.376	0.917	-0.239	-4.917	9.574E-07	*
69	Right_CA4_body	Hippocampus segmentation volume	0.121	1.018	0.358	1.003	-0.235	-4.894	1.073E-06	*
70	Right_fimbria	Hippocampus segmentation volume	0.076	1.061	0.240	0.996	-0.159	-3.303	9.763E-04	
71	Right_CA3_head	Hippocampus segmentation volume	0.108	0.978	0.271	0.897	-0.174	-3.586	3.439E-04	
72	Right_HATA	Hippocampus segmentation volume	0.192	1.018	0.489	0.885	-0.311	-6.396	2.017E-10	*
73	Left_AV	Thalamus segmentation volume	0.063	1.074	0.249	1.014	-0.179	-3.704	2.188E-04	
74	Left_CeM	Thalamus segmentation volume	0.029	1.080	0.334	1.067	-0.284	-5.923	3.773E-09	*
75	Left_CL	Thalamus segmentation volume	0.025	0.927	0.137	0.868	-0.124	-2.566	1.036E-02	
76	Left_CM	Thalamus segmentation volume	0.009	1.032	0.287	0.930	-0.283	-5.835	6.341E-09	*
77	Left_LD	Thalamus segmentation volume	0.161	0.920	0.286	0.934	-0.135	-2.813	4.954E-03	
78	Left_LGN	Thalamus segmentation volume	0.083	1.029	0.315	0.999	-0.229	-4.759	2.094E-06	*
79	Left_LP	Thalamus segmentation volume	0.136	0.990	0.237	0.984	-0.102	-2.132	3.312E-02	
80	Left_L_Sg	Thalamus segmentation volume	-0.026	1.005	0.109	0.947	-0.138	-2.863	4.248E-03	
81	Left_MDl	Thalamus segmentation volume	0.187	1.024	0.314	1.163	-0.116	-2.448	1.444E-02	
82	Left_MDm	Thalamus segmentation volume	0.247	1.063	0.430	1.158	-0.165	-3.467	5.380E-04	
83	Left_MGN	Thalamus segmentation volume	0.040	1.121	0.250	1.027	-0.195	-4.031	5.788E-05	*
84	Left_MV_Re_	Thalamus segmentation volume	0.066	0.990	0.376	1.018	-0.308	-6.446	1.463E-10	*
85	Left_Pc	Thalamus segmentation volume	0.099	1.068	0.283	1.063	-0.173	-3.612	3.117E-04	
86	Left_Pf	Thalamus segmentation volume	0.058	0.954	0.201	0.842	-0.159	-3.264	1.118E-03	
87	Left_Pt	Thalamus segmentation volume	0.077	1.079	0.269	0.927	-0.191	-3.920	9.163E-05	*
88	Left_PuA	Thalamus segmentation volume	0.137	1.107	0.387	1.111	-0.225	-4.704	2.737E-06	*
89	Left_PuI	Thalamus segmentation volume	-0.034	1.048	0.246	1.022	-0.271	-5.628	2.099E-08	*
90	Left_PuL	Thalamus segmentation volume	-0.062	1.120	0.012	1.177	-0.064	-1.350	1.770E-01	
91	Left_PuM	Thalamus segmentation volume	0.057	1.108	0.360	1.097	-0.275	-5.736	1.133E-08	*
92	Left_VA	Thalamus segmentation volume	0.049	1.001	0.223	1.023	-0.171	-3.587	3.432E-04	
93	Left_VAmc	Thalamus segmentation volume	-0.020	1.011	0.234	0.976	-0.256	-5.316	1.193E-07	*
94	Left_VLa	Thalamus segmentation volume	0.114	1.038	0.238	1.008	-0.121	-2.514	1.202E-02	
95	Left_VLp	Thalamus segmentation volume	0.118	1.046	0.268	0.992	-0.147	-3.043	2.379E-03	
96	Left_VM	Thalamus segmentation volume	0.085	0.967	0.254	0.928	-0.178	-3.698	2.237E-04	

97	Left_VPL	Thalamus segmentation volume	0.069	1.030	0.253	1.008	-0.180	-3.751	1.812E-04	
98	Right_AV	Thalamus segmentation volume	0.124	1.042	0.235	1.050	-0.106	-2.211	2.719E-02	
99	Right_CeM	Thalamus segmentation volume	0.046	1.106	0.313	1.083	-0.243	-5.068	4.434E-07	*
100	Right_CL	Thalamus segmentation volume	0.038	0.975	0.137	0.930	-0.103	-2.139	3.256E-02	
101	Right_CM	Thalamus segmentation volume	0.049	1.018	0.294	0.925	-0.253	-5.219	2.000E-07	*
102	Right_LD	Thalamus segmentation volume	0.211	0.938	0.327	0.926	-0.125	-2.596	9.516E-03	
103	Right_LGN	Thalamus segmentation volume	0.134	1.133	0.308	1.092	-0.157	-3.261	1.130E-03	
104	Right_LP	Thalamus segmentation volume	0.153	1.031	0.270	1.051	-0.113	-2.353	1.871E-02	
105	Right_L_Sg	Thalamus segmentation volume	-0.051	1.018	0.142	0.955	-0.195	-4.046	5.432E-05	*
106	Right_MDl	Thalamus segmentation volume	0.236	1.046	0.302	1.131	-0.060	-1.267	2.053E-01	
107	Right_MDm	Thalamus segmentation volume	0.284	1.071	0.417	1.172	-0.118	-2.498	1.257E-02	
108	Right_MGN	Thalamus segmentation volume	0.069	1.115	0.265	1.045	-0.181	-3.751	1.813E-04	
109	Right_MV_Re_	Thalamus segmentation volume	0.085	1.087	0.373	1.047	-0.270	-5.608	2.362E-08	*
110	Right_Pc	Thalamus segmentation volume	0.129	1.028	0.339	1.052	-0.202	-4.221	2.552E-05	*
111	Right_Pf	Thalamus segmentation volume	0.038	0.958	0.189	0.878	-0.164	-3.382	7.345E-04	
112	Right_Pt	Thalamus segmentation volume	0.143	1.041	0.339	0.953	-0.196	-4.055	5.228E-05	*
113	Right_PuA	Thalamus segmentation volume	0.196	1.039	0.338	1.038	-0.137	-2.861	4.277E-03	
114	Right_PuI	Thalamus segmentation volume	0.025	1.013	0.196	0.985	-0.171	-3.567	3.709E-04	
115	Right_PuL	Thalamus segmentation volume	-0.025	1.069	0.026	1.005	-0.048	-1.003	3.162E-01	
116	Right_PuM	Thalamus segmentation volume	0.128	1.027	0.319	1.020	-0.186	-3.887	1.052E-04	*
117	Right_VA	Thalamus segmentation volume	0.060	1.042	0.231	1.035	-0.165	-3.438	5.987E-04	
118	Right_VAmc	Thalamus segmentation volume	0.000	1.040	0.255	0.973	-0.253	-5.233	1.859E-07	*
119	Right_VLa	Thalamus segmentation volume	0.149	1.063	0.262	1.065	-0.107	-2.225	2.620E-02	
120	Right_VLp	Thalamus segmentation volume	0.160	1.066	0.271	1.066	-0.104	-2.173	2.987E-02	
121	Right_VM	Thalamus segmentation volume	0.107	1.011	0.236	0.992	-0.129	-2.691	7.187E-03	
122	Right_VPL	Thalamus segmentation volume	0.115	0.995	0.239	1.029	-0.123	-2.570	1.025E-02	
123	Medulla	Brain Stem segmentation volume	-0.108	0.952	0.122	0.917	-0.246	-5.111	3.529E-07	*
124	Pons	Brain Stem segmentation volume	-0.059	0.950	0.393	0.909	-0.486	-10.095	0.000E+00	*
125	SCP	Brain Stem segmentation volume	-0.030	1.010	0.233	0.954	-0.268	-5.546	3.353E-08	*
126	Midbrain	Brain Stem segmentation volume	-0.092	1.009	0.371	0.973	-0.467	-9.701	0.000E+00	*
127	Left_bankssts_volume	Cortical volume	0.169	0.979	0.080	1.028	0.089	1.866	6.225E-02	
128	Left_caudalanteriorcingulate_volume	Cortical volume	0.146	0.948	0.030	1.034	0.117	2.474	1.346E-02	
129	Left_caudalmiddlefrontal_volume	Cortical volume	0.314	0.955	-0.066	1.075	0.374	7.912	4.330E-15	*

130	Left_cuneus_volume	Cortical volume	0.121	0.939	0.052	0.974	0.073	1.521	1.284E-01	
131	Left_entorhinal_volume	Cortical volume	0.045	0.997	0.159	0.963	-0.116	-2.413	1.594E-02	
132	Left_fusiform_volume	Cortical volume	0.286	1.071	0.210	1.039	0.072	1.496	1.349E-01	
133	Left_inferiorparietal_volume	Cortical volume	0.267	0.995	0.066	1.016	0.200	4.188	2.943E-05	*
134	Left_inferiortemporal_volume	Cortical volume	0.251	1.050	0.183	1.029	0.065	1.362	1.733E-01	
135	Left_isthmuscingulate_volume	Cortical volume	0.155	0.888	0.001	0.965	0.166	3.495	4.850E-04	
136	Left_lateraloccipital_volume	Cortical volume	0.275	0.978	0.141	1.007	0.136	2.841	4.552E-03	
137	Left_lateralorbitofrontal_volume	Cortical volume	0.368	1.003	0.009	1.046	0.350	7.347	3.036E-13	*
138	Left_lingual_volume	Cortical volume	0.160	0.989	0.158	1.017	0.002	0.045	9.643E-01	
139	Left_medialorbitofrontal_volume	Cortical volume	0.177	0.984	-0.017	1.028	0.192	4.036	5.671E-05	*
140	Left_middletemporal_volume	Cortical volume	0.295	1.076	0.115	1.065	0.168	3.496	4.842E-04	
141	Left_parahippocampal_volume	Cortical volume	0.066	0.705	0.189	0.682	-0.177	-3.671	2.487E-04	
142	Left_paracentral_volume	Cortical volume	0.285	0.948	-0.007	1.008	0.299	6.282	4.175E-10	*
143	Left_parsopercularis_volume	Cortical volume	0.275	0.976	-0.037	0.994	0.316	6.614	4.903E-11	*
144	Left_parsorbitalis_volume	Cortical volume	0.249	1.053	-0.006	1.045	0.243	5.061	4.589E-07	*
145	Left_parstriangularis_volume	Cortical volume	0.265	0.952	-0.042	1.031	0.309	6.511	9.589E-11	*
146	Left_pericalcarine_volume	Cortical volume	0.033	0.958	-0.014	0.980	0.048	1.013	3.111E-01	
147	Left_postcentral_volume	Cortical volume	0.387	0.969	0.074	1.004	0.317	6.645	3.970E-11	*
148	Left_posteriorcingulate_volume	Cortical volume	0.253	0.960	0.030	1.196	0.206	4.410	1.094E-05	*
149	Left_precentral_volume	Cortical volume	0.415	0.968	0.098	1.009	0.320	6.710	2.584E-11	*
150	Left_precuneus_volume	Cortical volume	0.281	0.986	-0.018	1.018	0.298	6.250	5.089E-10	*
151	Left_rostralanteriorcingulate_volume	Cortical volume	0.215	0.871	0.035	0.965	0.195	4.120	3.951E-05	*
152	Left_rostralmiddlefrontal_volume	Cortical volume	0.278	0.999	-0.021	0.964	0.305	6.340	2.879E-10	*
153	Left_superiorfrontal_volume	Cortical volume	0.485	0.973	-0.018	1.110	0.483	10.225	0.000E+00	*
154	Left_superiorparietal_volume	Cortical volume	0.251	1.014	-0.064	1.000	0.312	6.507	9.845E-11	*
155	Left_superiortemporal_volume	Cortical volume	0.333	0.993	0.190	1.063	0.140	2.942	3.306E-03	
156	Left_supramarginal_volume	Cortical volume	0.293	0.971	0.096	0.932	0.208	4.310	1.716E-05	*
157	Left_frontalpole_volume	Cortical volume	0.147	1.022	-0.003	1.038	0.146	3.043	2.378E-03	
158	Left_temporalpole_volume	Cortical volume	0.010	0.982	0.051	0.987	-0.042	-0.874	3.823E-01	
159	Left_transversetemporal_volume	Cortical volume	0.261	0.979	0.045	1.076	0.209	4.413	1.078E-05	*
160	Left_insula_volume	Cortical volume	0.288	0.736	0.157	0.759	0.175	3.669	2.501E-04	
161	Right_bankssts_volume	Cortical volume	0.232	1.035	0.084	1.112	0.137	2.891	3.890E-03	
162	Right_caudalanteriorcingulate_volume	Cortical volume	0.119	0.978	0.028	1.099	0.088	1.852	6.413E-02	

163	Right_caudalmiddlefrontal_volume	Cortical volume	0.275	0.928	-0.095	0.981	0.388	8.145	6.661E-16	*
164	Right_cuneus_volume	Cortical volume	0.160	0.917	0.062	0.951	0.104	2.187	2.889E-02	
165	Right_entorhinal_volume	Cortical volume	0.039	0.987	0.110	0.991	-0.072	-1.500	1.337E-01	
166	Right_fusiform_volume	Cortical volume	0.285	0.993	0.217	1.026	0.067	1.413	1.578E-01	
167	Right_inferiorparietal_volume	Cortical volume	0.261	1.007	0.163	0.996	0.098	2.038	4.170E-02	
168	Right_inferiortemporal_volume	Cortical volume	0.279	1.011	0.164	1.008	0.114	2.371	1.786E-02	
169	Right_isthmuscingulate_volume	Cortical volume	0.183	0.985	0.034	1.061	0.146	3.069	2.181E-03	
170	Right_lateraloccipital_volume	Cortical volume	0.266	0.984	0.145	1.039	0.120	2.510	1.215E-02	
171	Right_lateralorbitofrontal_volume	Cortical volume	0.323	1.064	0.000	1.077	0.301	6.299	3.747E-10	*
172	Right_lingual_volume	Cortical volume	0.142	1.020	0.153	0.948	-0.011	-0.218	8.277E-01	
173	Right_medialorbitofrontal_volume	Cortical volume	0.295	0.879	0.061	0.958	0.255	5.381	8.378E-08	*
174	Right_middletemporal_volume	Cortical volume	0.294	1.023	0.121	1.042	0.167	3.492	4.910E-04	
175	Right_parahippocampal_volume	Cortical volume	0.057	1.033	0.321	0.967	-0.264	-5.474	5.002E-08	*
176	Right_paracentral_volume	Cortical volume	0.321	0.973	0.036	0.976	0.293	6.120	1.143E-09	*
177	Right_parsopercularis_volume	Cortical volume	0.273	0.997	-0.010	1.049	0.277	5.808	7.424E-09	*
178	Right_parsorbitalis_volume	Cortical volume	0.212	1.061	-0.001	1.114	0.196	4.104	4.244E-05	*
179	Right_parstriangularis_volume	Cortical volume	0.241	1.039	-0.033	1.040	0.264	5.509	4.113E-08	*
180	Right_pericalcarine_volume	Cortical volume	0.025	0.971	-0.036	0.988	0.063	1.315	1.887E-01	
181	Right_postcentral_volume	Cortical volume	0.328	0.993	0.010	1.017	0.317	6.625	4.539E-11	*
182	Right_posteriorcingulate_volume	Cortical volume	0.312	1.017	0.051	1.319	0.222	4.764	2.050E-06	*
183	Right_precentral_volume	Cortical volume	0.376	1.042	-0.022	1.014	0.387	8.047	1.554E-15	*
184	Right_precuneus_volume	Cortical volume	0.314	1.043	-0.014	1.048	0.314	6.553	7.290E-11	*
185	Right_rostralanteriorcingulate_volume	Cortical volume	0.169	0.992	0.108	1.055	0.060	1.255	2.096E-01	
186	Right_rostralmiddlefrontal_volume	Cortical volume	0.233	1.023	0.009	0.955	0.226	4.672	3.206E-06	*
187	Right_superiorfrontal_volume	Cortical volume	0.421	1.031	-0.023	1.027	0.432	9.009	0.000E+00	*
188	Right_superiorparietal_volume	Cortical volume	0.282	1.015	-0.022	0.995	0.303	6.300	3.718E-10	*
189	Right_superiortemporal_volume	Cortical volume	0.323	1.097	0.158	1.093	0.150	3.136	1.741E-03	
190	Right_supramarginal_volume	Cortical volume	0.310	1.039	0.001	1.044	0.297	6.203	6.819E-10	*
191	Right_frontalpole_volume	Cortical volume	0.182	1.029	0.043	1.012	0.136	2.839	4.577E-03	
192	Right_temporalpole_volume	Cortical volume	-0.010	0.980	0.067	0.960	-0.079	-1.645	1.002E-01	
193	Right_transversetemporal_volume	Cortical volume	0.310	0.998	0.099	1.047	0.206	4.325	1.609E-05	*
194	Right_insula_volume	Cortical volume	0.251	0.694	0.178	0.746	0.101	2.117	3.437E-02	
195	Left_bankssts_area	Cortical Surface Area	0.127	1.028	0.037	1.079	0.085	1.792	7.324E-02	

196	Left_caudalanteriorcingulate_area	Cortical Surface Area	0.141	0.921	-0.016	0.997	0.164	3.449	5.764E-04	
197	Left_caudalmiddlefrontal_area	Cortical Surface Area	0.210	0.972	-0.120	1.057	0.325	6.857	9.547E-12	*
198	Left_cuneus_area	Cortical Surface Area	0.120	0.979	0.046	0.983	0.075	1.570	1.166E-01	
199	Left_entorhinal_area	Cortical Surface Area	0.059	0.922	0.105	0.887	-0.052	-1.070	2.846E-01	
200	Left_fusiform_area	Cortical Surface Area	0.177	1.083	0.119	1.057	0.054	1.128	2.595E-01	
201	Left_inferiorparietal_area	Cortical Surface Area	0.162	1.033	-0.001	1.042	0.156	3.266	1.111E-03	
202	Left_inferiortemporal_area	Cortical Surface Area	0.137	1.065	0.058	1.111	0.072	1.517	1.295E-01	
203	Left_isthmuscingulate_area	Cortical Surface Area	0.063	0.839	-0.087	1.080	0.155	3.322	9.119E-04	
204	Left_lateraloccipital_area	Cortical Surface Area	0.165	0.988	0.061	0.988	0.105	2.200	2.794E-02	
205	Left_lateralorbitofrontal_area	Cortical Surface Area	0.223	0.997	-0.060	1.008	0.282	5.888	4.624E-09	*
206	Left_lingual_area	Cortical Surface Area	0.082	1.011	0.087	1.012	-0.005	-0.105	9.165E-01	
207	Left_medialorbitofrontal_area	Cortical Surface Area	0.096	1.025	-0.051	1.060	0.141	2.956	3.157E-03	
208	Left_middletemporal_area	Cortical Surface Area	0.151	1.090	-0.020	1.114	0.155	3.248	1.182E-03	
209	Left_parahippocampal_area	Cortical Surface Area	0.006	0.698	0.132	0.571	-0.197	-4.020	6.056E-05	*
210	Left_paracentral_area	Cortical Surface Area	0.132	0.952	-0.066	0.967	0.206	4.298	1.814E-05	*
211	Left_parsopercularis_area	Cortical Surface Area	0.189	0.986	-0.094	1.023	0.282	5.900	4.330E-09	*
212	Left_parsorbitalis_area	Cortical Surface Area	0.195	1.024	-0.041	1.015	0.232	4.840	1.408E-06	*
213	Left_parstriangularis_area	Cortical Surface Area	0.167	0.951	-0.091	1.040	0.259	5.470	5.112E-08	*
214	Left_pericalcarine_area	Cortical Surface Area	0.065	0.972	0.047	0.958	0.018	0.378	7.056E-01	
215	Left_postcentral_area	Cortical Surface Area	0.232	1.003	-0.046	0.988	0.280	5.827	6.654E-09	*
216	Left_posteriorcingulate_area	Cortical Surface Area	0.162	0.953	-0.024	1.459	0.151	3.312	9.441E-04	
217	Left_precentral_area	Cortical Surface Area	0.220	0.977	0.000	0.990	0.224	4.682	3.054E-06	*
218	Left_precuneus_area	Cortical Surface Area	0.151	0.995	-0.087	1.034	0.234	4.905	1.016E-06	*
219	Left_rostralanteriorcingulate_area	Cortical Surface Area	0.197	0.904	0.020	0.947	0.192	4.026	5.895E-05	*
220	Left_rostralmiddlefrontal_area	Cortical Surface Area	0.150	1.027	-0.110	1.001	0.257	5.350	9.915E-08	*
221	Left_superiorfrontal_area	Cortical Surface Area	0.284	0.994	-0.113	1.070	0.384	8.084	1.110E-15	*
222	Left_superiorparietal_area	Cortical Surface Area	0.131	1.039	-0.120	1.016	0.244	5.072	4.341E-07	*
223	Left_superiortemporal_area	Cortical Surface Area	0.212	0.991	-0.013	1.074	0.217	4.570	5.199E-06	*
224	Left_supramarginal_area	Cortical Surface Area	0.172	0.986	-0.006	0.993	0.180	3.750	1.825E-04	
225	Left_frontalpole_area	Cortical Surface Area	0.065	1.027	-0.078	1.038	0.139	2.897	3.818E-03	
226	Left_temporalpole_area	Cortical Surface Area	0.013	0.962	-0.010	1.030	0.022	0.468	6.397E-01	
227	Left_transversetemporal_area	Cortical Surface Area	0.193	0.989	-0.065	1.090	0.248	5.223	1.962E-07	*
228	Left_insula_area	Cortical Surface Area	0.182	0.779	0.088	0.788	0.121	2.522	1.177E-02	

229	Right_bankssts_area	Cortical Surface Area	0.180	1.028	0.010	1.107	0.159	3.343	8.458E-04	
230	Right_caudalanteriorcingulate_area	Cortical Surface Area	0.091	0.963	-0.027	1.087	0.115	2.436	1.496E-02	
231	Right_caudalmiddlefrontal_area	Cortical Surface Area	0.168	0.962	-0.160	0.993	0.336	7.039	2.727E-12	*
232	Right_cuneus_area	Cortical Surface Area	0.137	0.937	0.015	0.929	0.130	2.718	6.630E-03	
233	Right_entorhinal_area	Cortical Surface Area	0.004	0.932	0.037	0.998	-0.034	-0.707	4.795E-01	
234	Right_fusiform_area	Cortical Surface Area	0.171	1.045	0.086	1.076	0.079	1.659	9.731E-02	
235	Right_inferiorparietal_area	Cortical Surface Area	0.164	1.028	0.046	1.034	0.115	2.395	1.671E-02	
236	Right_inferiortemporal_area	Cortical Surface Area	0.198	1.033	0.016	1.081	0.172	3.604	3.217E-04	
237	Right_isthmuscingulate_area	Cortical Surface Area	0.072	1.001	-0.107	1.246	0.158	3.379	7.439E-04	
238	Right_lateraloccipital_area	Cortical Surface Area	0.143	0.999	0.055	1.009	0.088	1.830	6.748E-02	
239	Right_lateralorbitofrontal_area	Cortical Surface Area	0.185	1.041	-0.098	1.050	0.271	5.651	1.849E-08	*
240	Right_lingual_area	Cortical Surface Area	0.093	1.000	0.080	0.935	0.013	0.269	7.880E-01	
241	Right_medialorbitofrontal_area	Cortical Surface Area	0.202	0.935	0.030	1.013	0.176	3.709	2.146E-04	
242	Right_middletemporal_area	Cortical Surface Area	0.196	1.041	-0.031	1.079	0.214	4.487	7.660E-06	*
243	Right_parahippocampal_area	Cortical Surface Area	-0.024	1.027	0.199	0.919	-0.229	-4.713	2.625E-06	*
244	Right_paracentral_area	Cortical Surface Area	0.153	0.983	-0.028	0.976	0.184	3.845	1.246E-04	*
245	Right_parsopercularis_area	Cortical Surface Area	0.180	0.996	-0.062	1.039	0.238	4.985	6.793E-07	*
246	Right_parsorbitalis_area	Cortical Surface Area	0.139	1.024	-0.044	1.042	0.177	3.698	2.234E-04	
247	Right_parstriangularis_area	Cortical Surface Area	0.164	1.027	-0.075	1.011	0.235	4.885	1.126E-06	*
248	Right_pericalcarine_area	Cortical Surface Area	0.094	0.949	0.023	0.909	0.076	1.582	1.137E-01	
249	Right_postcentral_area	Cortical Surface Area	0.211	1.045	-0.101	1.028	0.301	6.272	4.419E-10	*
250	Right_posteriorcingulate_area	Cortical Surface Area	0.201	1.011	-0.038	1.524	0.185	4.043	5.488E-05	*
251	Right_precentral_area	Cortical Surface Area	0.197	0.991	-0.100	1.030	0.294	6.165	8.625E-10	*
252	Right_precuneus_area	Cortical Surface Area	0.179	1.039	-0.095	1.050	0.262	5.469	5.140E-08	*
253	Right_rostralanteriorcingulate_area	Cortical Surface Area	0.178	0.967	0.086	0.992	0.093	1.952	5.111E-02	
254	Right_rostralmiddlefrontal_area	Cortical Surface Area	0.134	1.063	-0.064	0.994	0.192	3.970	7.460E-05	*
255	Right_superiorfrontal_area	Cortical Surface Area	0.229	1.063	-0.107	1.014	0.323	6.706	2.654E-11	*
256	Right_superiorparietal_area	Cortical Surface Area	0.164	1.052	-0.087	0.999	0.245	5.083	4.087E-07	*
257	Right_superiortemporal_area	Cortical Surface Area	0.184	1.092	-0.019	1.084	0.187	3.891	1.036E-04	*
258	Right_supramarginal_area	Cortical Surface Area	0.192	1.014	-0.090	1.029	0.277	5.779	8.803E-09	*
259	Right_frontalpole_area	Cortical Surface Area	0.114	1.018	-0.028	1.025	0.139	2.907	3.697E-03	
260	Right_temporalpole_area	Cortical Surface Area	-0.052	0.984	-0.043	0.958	-0.010	-0.198	8.430E-01	
261	Right_transversetemporal_area	Cortical Surface Area	0.202	0.994	-0.049	1.030	0.248	5.205	2.159E-07	*

262	Right_insula_area	Cortical Surface Area	0.141	0.689	0.101	0.737	0.056	1.182	2.373E-01	
263	Left_bankssts_thickness	Cortical Thickness	0.179	1.088	0.210	1.069	-0.029	-0.594	5.524E-01	
264	Left_caudalanteriorcingulate_thickness	Cortical Thickness	0.048	1.016	0.126	0.977	-0.078	-1.616	1.063E-01	
265	Left_caudalmiddlefrontal_thickness	Cortical Thickness	0.362	1.056	0.215	1.082	0.138	2.879	4.037E-03	
266	Left_cuneus_thickness	Cortical Thickness	0.079	0.962	0.027	1.044	0.052	1.093	2.747E-01	
267	Left_entorhinal_thickness	Cortical Thickness	0.069	0.954	0.091	0.954	-0.023	-0.474	6.352E-01	
268	Left_fusiform_thickness	Cortical Thickness	0.318	1.058	0.344	1.059	-0.025	-0.523	6.009E-01	
269	Left_inferiorparietal_thickness	Cortical Thickness	0.311	1.086	0.213	1.099	0.090	1.881	6.008E-02	
270	Left_inferiortemporal_thickness	Cortical Thickness	0.310	1.021	0.366	0.991	-0.055	-1.151	2.499E-01	
271	Left_isthmuscingulate_thickness	Cortical Thickness	0.181	0.976	0.122	0.954	0.062	1.285	1.991E-01	
272	Left_lateraloccipital_thickness	Cortical Thickness	0.216	1.061	0.153	1.094	0.058	1.210	2.265E-01	
273	Left_lateralorbitofrontal_thickness	Cortical Thickness	0.277	1.012	0.217	1.033	0.059	1.239	2.155E-01	
274	Left_lingual_thickness	Cortical Thickness	0.201	1.037	0.163	1.049	0.036	0.753	4.515E-01	
275	Left_medialorbitofrontal_thickness	Cortical Thickness	0.161	1.004	0.119	0.999	0.041	0.863	3.883E-01	
276	Left_middletemporal_thickness	Cortical Thickness	0.319	1.148	0.315	1.145	0.004	0.078	9.382E-01	
277	Left_parahippocampal_thickness	Cortical Thickness	0.109	0.983	0.157	1.004	-0.048	-1.002	3.164E-01	
278	Left_paracentral_thickness	Cortical Thickness	0.299	1.056	0.097	1.095	0.188	3.939	8.504E-05	*
279	Left_parsopercularis_thickness	Cortical Thickness	0.341	1.064	0.222	1.022	0.114	2.364	1.819E-02	
280	Left_parsorbitalis_thickness	Cortical Thickness	0.227	0.958	0.137	1.075	0.089	1.875	6.097E-02	
281	Left_parstriangularis_thickness	Cortical Thickness	0.327	0.954	0.174	1.021	0.154	3.246	1.193E-03	
282	Left_pericalcarine_thickness	Cortical Thickness	-0.014	0.980	-0.050	0.964	0.037	0.776	4.379E-01	
283	Left_postcentral_thickness	Cortical Thickness	0.300	1.067	0.236	1.012	0.061	1.272	2.036E-01	
284	Left_posteriorcingulate_thickness	Cortical Thickness	0.194	0.989	0.115	1.093	0.076	1.606	1.085E-01	
285	Left_precentral_thickness	Cortical Thickness	0.335	1.104	0.163	0.986	0.164	3.384	7.297E-04	
286	Left_precuneus_thickness	Cortical Thickness	0.308	1.046	0.158	1.097	0.140	2.927	3.468E-03	
287	Left_rostralanteriorcingulate_thickness	Cortical Thickness	0.046	0.972	0.111	0.952	-0.068	-1.414	1.576E-01	
288	Left_rostralmiddlefrontal_thickness	Cortical Thickness	0.345	1.004	0.264	1.033	0.079	1.658	9.751E-02	
289	Left_superiorfrontal_thickness	Cortical Thickness	0.408	1.036	0.255	1.076	0.145	3.043	2.375E-03	
290	Left_superiorparietal_thickness	Cortical Thickness	0.276	1.071	0.139	1.091	0.127	2.650	8.113E-03	
291	Left_superiortemporal_thickness	Cortical Thickness	0.274	1.094	0.397	1.112	-0.112	-2.336	1.962E-02	
292	Left_supramarginal_thickness	Cortical Thickness	0.348	1.089	0.275	1.092	0.067	1.404	1.604E-01	
293	Left_frontalpole_thickness	Cortical Thickness	0.193	1.002	0.141	1.023	0.051	1.065	2.870E-01	
294	Left_temporalpole_thickness	Cortical Thickness	0.024	0.971	0.133	1.002	-0.111	-2.316	2.064E-02	

295	Left_transversetemporal_thickness	Cortical Thickness	0.162	1.028	0.200	1.037	-0.037	-0.766	4.439E-01	
296	Left_insula_thickness	Cortical Thickness	0.225	0.984	0.148	1.029	0.077	1.614	1.066E-01	
297	Right_bankssts_thickness	Cortical Thickness	0.151	0.974	0.192	1.052	-0.040	-0.848	3.966E-01	
298	Right_caudalanteriorcingulate_thickness	Cortical Thickness	0.037	0.996	0.134	1.031	-0.096	-2.009	4.468E-02	
299	Right_caudalmiddlefrontal_thickness	Cortical Thickness	0.321	1.068	0.203	1.114	0.108	2.263	2.374E-02	
300	Right_cuneus_thickness	Cortical Thickness	0.103	0.973	0.065	1.010	0.039	0.821	4.119E-01	
301	Right_entorhinal_thickness	Cortical Thickness	0.094	0.974	0.100	1.017	-0.006	-0.129	8.971E-01	
302	Right_fusiform_thickness	Cortical Thickness	0.313	1.028	0.409	1.000	-0.094	-1.965	4.959E-02	
303	Right_inferiorparietal_thickness	Cortical Thickness	0.236	1.062	0.262	1.052	-0.024	-0.506	6.126E-01	
304	Right_inferiortemporal_thickness	Cortical Thickness	0.272	1.096	0.351	1.058	-0.074	-1.537	1.246E-01	
305	Right_isthmuscingulate_thickness	Cortical Thickness	0.194	0.976	0.210	0.990	-0.016	-0.332	7.402E-01	
306	Right_lateraloccipital_thickness	Cortical Thickness	0.210	1.005	0.154	1.052	0.054	1.139	2.549E-01	
307	Right_lateralorbitofrontal_thickness	Cortical Thickness	0.232	0.986	0.233	1.040	-0.001	-0.017	9.862E-01	
308	Right_lingual_thickness	Cortical Thickness	0.139	0.973	0.166	0.967	-0.028	-0.588	5.563E-01	
309	Right_medialorbitofrontal_thickness	Cortical Thickness	0.181	0.998	0.108	1.040	0.072	1.506	1.323E-01	
310	Right_middletemporal_thickness	Cortical Thickness	0.222	1.084	0.314	1.150	-0.083	-1.734	8.304E-02	
311	Right_parahippocampal_thickness	Cortical Thickness	0.121	1.022	0.234	1.028	-0.110	-2.300	2.153E-02	
312	Right_paracentral_thickness	Cortical Thickness	0.333	1.085	0.142	1.075	0.177	3.687	2.335E-04	
313	Right_parsopercularis_thickness	Cortical Thickness	0.340	1.037	0.192	1.032	0.143	2.976	2.956E-03	
314	Right_parsorbitalis_thickness	Cortical Thickness	0.216	1.040	0.168	1.092	0.046	0.956	3.394E-01	
315	Right_parstriangularis_thickness	Cortical Thickness	0.273	1.017	0.169	1.149	0.096	2.033	4.218E-02	
316	Right_pericalcarine_thickness	Cortical Thickness	-0.068	0.964	-0.038	1.017	-0.031	-0.645	5.187E-01	
317	Right_postcentral_thickness	Cortical Thickness	0.193	1.065	0.182	0.992	0.011	0.218	8.275E-01	
318	Right_posteriorcingulate_thickness	Cortical Thickness	0.221	0.965	0.202	1.072	0.018	0.385	7.003E-01	
319	Right_precentral_thickness	Cortical Thickness	0.299	1.125	0.101	1.007	0.186	3.825	1.349E-04	*
320	Right_precuneus_thickness	Cortical Thickness	0.325	1.034	0.170	1.083	0.146	3.072	2.156E-03	
321	Right_rostralanteriorcingulate_thickness	Cortical Thickness	0.000	1.082	0.001	1.106	-0.001	-0.026	9.795E-01	
322	Right_rostralmiddlefrontal_thickness	Cortical Thickness	0.265	1.036	0.245	1.036	0.019	0.405	6.858E-01	
323	Right_superiorfrontal_thickness	Cortical Thickness	0.407	1.051	0.250	1.070	0.149	3.114	1.874E-03	
324	Right_superiorparietal_thickness	Cortical Thickness	0.276	1.034	0.159	1.062	0.112	2.337	1.957E-02	
325	Right_superiortemporal_thickness	Cortical Thickness	0.271	1.075	0.319	1.090	-0.045	-0.941	3.468E-01	
326	Right_supramarginal_thickness	Cortical Thickness	0.299	1.098	0.240	1.135	0.053	1.108	2.679E-01	
327	Right_frontalpole_thickness	Cortical Thickness	0.192	1.008	0.173	1.017	0.019	0.401	6.882E-01	

328	Right_temporalpole_thickness	Cortical Thickness	0.047	0.979	0.173	1.036	-0.124	-2.612	9.074E-03
329	Right_transversetemporal_thickness	Cortical Thickness	0.216	1.012	0.231	1.056	-0.015	-0.311	7.555E-01
330	Right_insula_thickness	Cortical Thickness	0.273	0.983	0.189	1.008	0.084	1.748	8.062E-02

Note: * two-sided $p<0.05$ (derived from two sample *t* test) after Family Wise Error (FWE) correction.

Supplementary Table 6. Difference of volume of striatum between the subtype1 and subtype2 in a medication-naïve subsample.

Region	Morphological measure	Subtype1(*n*=445)		Subtype2(*n*=309)		Subtype1 vs. Subtype2		
		Mean	SD	Mean	SD	Cohen's d	*t*	*p*
Left_Caudate	Subcortical volume	-0.284	1.003	0.299	1.089	-0.557	-7.617	0.000 *
Left_Putamen	Subcortical volume	-0.197	1.069	0.230	1.056	-0.402	-5.442	0.000 *
Right_Caudate	Subcortical volume	-0.274	1.048	0.251	1.148	-0.478	-6.538	0.000 *
Right_Putamen	Subcortical volume	-0.192	1.025	0.203	1.084	-0.374	-5.104	0.000 *

Note: * two-sided $p<0.05$ (derived from two sample *t* test) after Family Wise Error (FWE) correction.

Supplementary Table 7. AAL3 brain regions.

Feature ID	SuStaIn Feature	AAL3 ROI Name	AAL3 ROI Full Name
1	Hippocampus	lHIP	Left Hippocampus
1	Hippocampus	rHIP	Right Hippocampus
2	Parahippocampus	lPHG	Left Parahippocampal gyrus
2	Parahippocampus	rPHG	Right Parahippocampal gyrus
3	Amygdala	lAMYG	Left Amygdala
3	Amygdala	rAMYG	Right Amygdala
4	Caudate	lCAU	Left Caudate nucleus
4	Caudate	rCAU	Right Caudate nucleus
5	Putamen	lPUT	Left Lenticular nucleus-Putamen
5	Putamen	rPUT	Right Lenticular nucleus-Putamen
6	Pallidum	lPAL	Left Lenticular nucleus-Pallidum
6	Pallidum	rPAL	Right Lenticular nucleus-Pallidum
7	Thalamus	ltAV	Left Thalamus-Anteroventral Nucleus
7	Thalamus	rtAV	Right Thalamus-Anteroventral Nucleus
7	Thalamus	ltLP	Left Lateral posterior
7	Thalamus	rtLP	Right Lateral posterior
7	Thalamus	ltVA	Left Ventral anterior
7	Thalamus	rtVA	Right Ventral anterior
7	Thalamus	ltVL	Left Ventral lateral
7	Thalamus	rtVL	Right Ventral lateral
7	Thalamus	ltVPL	Left Ventral posterolateral
7	Thalamus	rtVPL	Right Ventral posterolateral
7	Thalamus	ltIL	Left Intralaminar
7	Thalamus	rtIL	Right Intralaminar
7	Thalamus	ltMDm	Left Mediodorsal medial magnocellular
7	Thalamus	rtMDm	Right Mediodorsal medial magnocellular
7	Thalamus	ltMDl	Left Mediodorsal lateral parvocellular
7	Thalamus	rtMDl	Right Mediodorsal lateral parvocellular
7	Thalamus	ltLGN	Left Lateral geniculate
7	Thalamus	rtLGN	Right Lateral geniculate
7	Thalamus	ltMGN	Left Medial Geniculate

7	Thalamus	rtMGN	Right Medial Geniculate
7	Thalamus	ltPuA	Left Pulvinar anterior
7	Thalamus	rtPuA	Right Pulvinar anterior
7	Thalamus	ltPuM	Left Pulvinar medial
7	Thalamus	rtPuM	Right Pulvinar medial
7	Thalamus	ltPuL	Left Pulvinar lateral
7	Thalamus	rtPuL	Right Pulvinar lateral
7	Thalamus	ltPuI	Left Pulvinar inferior
7	Thalamus	rtPuI	Right Pulvinar inferior
8	Accumbens	lNacc	Left Nucleus accumbens
8	Accumbens	rNacc	Right Nucleus accumbens
9	Cingulate	lMCC	Left Middle cingulate & paracingulate gyri
9	Cingulate	rMCC	Right Middle cingulate & paracingulate gyri
9	Cingulate	lPCC	Left Posterior cingulate gyrus
9	Cingulate	rPCC	Right Posterior cingulate gyrus
9	Cingulate	lACCsub	Left Anterior cingulate cortex-subgenual
9	Cingulate	rACCsub	Right Anterior cingulate cortex-subgenual
9	Cingulate	lACCpre	Left Anterior cingulate cortex-pregenual
9	Cingulate	rACCpre	Right Anterior cingulate cortex-pregenual
9	Cingulate	lACCsup	Left Anterior cingulate cortex-supracallosal
9	Cingulate	rACCsup	Right Anterior cingulate cortex-supracallosal
10	Frontal Cortex	lSFG	Left Superior frontal gyrus-dorsolateral
10	Frontal Cortex	rSFG	Right Superior frontal gyrus-dorsolateral
10	Frontal Cortex	lMFG	Left Middle frontal gyrus
10	Frontal Cortex	rMFG	Right Middle frontal gyrus
10	Frontal Cortex	lIFGorb	Left IFG pars orbitalis
10	Frontal Cortex	rIFGorb	Right IFG pars orbitalis
10	Frontal Cortex	lROL	Left Rolandic operculum
10	Frontal Cortex	rROL	Right Rolandic operculum
10	Frontal Cortex	lOLF	Left Olfactory cortex
10	Frontal Cortex	rOLF	Right Olfactory cortex
10	Frontal Cortex	lSFGmedial	Left Superior frontal gyrus-medial
10	Frontal Cortex	rSFGmedial	Right Superior frontal gyrus-medial

10	Frontal Cortex	lPFCventmed	Left Superior frontal gyrus-medial orbital
10	Frontal Cortex	rPFCventmed	Right Superior frontal gyrus-medial orbital
10	Frontal Cortex	lREC	Left Gyrus rectus
10	Frontal Cortex	rREC	Right Gyrus rectus
10	Frontal Cortex	lOFCmed	Left Medial orbital gyrus
10	Frontal Cortex	rOFCmed	Right Medial orbital gyrus
10	Frontal Cortex	lOFCant	Left Anterior orbital gyrus
10	Frontal Cortex	rOFCant	Right Anterior orbital gyrus
10	Frontal Cortex	lOFCpost	Left Posterior orbital gyrus
10	Frontal Cortex	rOFCpost	Right Posterior orbital gyrus
10	Frontal Cortex	lOFClat	Left Lateral orbital gyrus
10	Frontal Cortex	rOFClat	Right Lateral orbital gyrus
11	Parietal Cortex	lSPG	Left Superior parietal gyrus
11	Parietal Cortex	rSPG	Right Superior parietal gyrus
11	Parietal Cortex	lIPG	Left Inferior parietal gyrus-excluding supramarginal and angular gyri
11	Parietal Cortex	rIPG	Right Inferior parietal gyrus-excluding supramarginal and angular gyri
11	Parietal Cortex	lSMG	Left SupraMarginal gyrus
11	Parietal Cortex	rSMG	Right SupraMarginal gyrus
11	Parietal Cortex	lANG	Left Angular gyrus
11	Parietal Cortex	rANG	Right Angular gyrus
11	Parietal Cortex	lPCUN	Left Precuneus
11	Parietal Cortex	rPCUN	Right Precuneus
11	Parietal Cortex	lPCL	Left Paracentral lobule
11	Parietal Cortex	rPCL	Right Paracentral lobule
12	Temporal Cortex	lFFG	Left Fusiform gyrus
12	Temporal Cortex	rFFG	Right Fusiform gyrus
12	Temporal Cortex	lHES	Left Heschls gyrus
12	Temporal Cortex	rHES	Right Heschls gyrus
12	Temporal Cortex	lSTG	Left Superior temporal gyrus
12	Temporal Cortex	rSTG	Right Superior temporal gyrus
12	Temporal Cortex	lTPOsup	Left Temporal pole: superior temporal gyrus
12	Temporal Cortex	rTPOsup	Right Temporal pole: superior temporal gyrus
12	Temporal Cortex	lMTG	Left Middle temporal gyrus

12	Temporal Cortex	rMTG	Right Middle temporal gyrus
12	Temporal Cortex	lTPOmid	Left Temporal pole: middle temporal gyrus
12	Temporal Cortex	rTPOmid	Right Temporal pole: middle temporal gyrus
12	Temporal Cortex	lITG	Left Inferior temporal gyrus
12	Temporal Cortex	rITG	Right Inferior temporal gyrus
13	Occipital Cortex	lCAL	Left Calcarine fissure and surrounding cortex
13	Occipital Cortex	rCAL	Right Calcarine fissure and surrounding cortex
13	Occipital Cortex	lCUN	Left Cuneus
13	Occipital Cortex	rCUN	Right Cuneus
13	Occipital Cortex	lLING	Left Lingual gyrus
13	Occipital Cortex	rLING	Right Lingual gyrus
13	Occipital Cortex	lSOG	Left Superior occipital gyrus
13	Occipital Cortex	rSOG	Right Superior occipital gyrus
13	Occipital Cortex	lMOG	Left Middle occipital gyrus
13	Occipital Cortex	rMOG	Right Middle occipital gyrus
13	Occipital Cortex	lIOG	Left Inferior occipital gyrus
13	Occipital Cortex	rIOG	Right Inferior occipital gyrus
14	Insula	lINS	Left Insula
14	Insula	rINS	Right Insula
15	Cerebellum	lCERCRU1	Left Crus I of cerebellar hemisphere
15	Cerebellum	rCERCRU1	Right Crus I of cerebellar hemisphere
15	Cerebellum	lCERCRU2	Left Crus II of cerebellar hemisphere
15	Cerebellum	rCERCRU2	Right Crus II of cerebellar hemisphere
15	Cerebellum	lCER3	Left Lobule III of cerebellar hemisphere
15	Cerebellum	rCER3	Right Lobule III of cerebellar hemisphere
15	Cerebellum	lCER4_5	Left Lobule IV-V of cerebellar hemisphere
15	Cerebellum	rCER4_5	Right Lobule IV-V of cerebellar hemisphere
15	Cerebellum	lCER6	Left Lobule VI of cerebellar hemisphere
15	Cerebellum	rCER6	Right Lobule VI of cerebellar hemisphere
15	Cerebellum	lCER7b	Left Lobule VIIB of cerebellar hemisphere
15	Cerebellum	rCER7b	Right Lobule VIIB of cerebellar hemisphere
15	Cerebellum	lCER8	Left Lobule VIII of cerebellar hemisphere
15	Cerebellum	rCER8	Right Lobule VIII of cerebellar hemisphere

15	Cerebellum	lCER9	Left Lobule IX of cerebellar hemisphere
15	Cerebellum	rCER9	Right Lobule IX of cerebellar hemisphere
15	Cerebellum	lCER10	Left Lobule X of cerebellar hemisphere
15	Cerebellum	rCER10	Right Lobule X of cerebellar hemisphere
15	Cerebellum	VER1_2	Lobule I-II of vermis
15	Cerebellum	VER3	Lobule III of vermis
15	Cerebellum	VER4_5	Lobule IV-V of vermis
15	Cerebellum	VER6	Lobule VI of vermis
15	Cerebellum	VER7	Lobule VII of vermis
15	Cerebellum	VER8	Lobule VIII of vermis
15	Cerebellum	VER9	Lobule IX of vermis
15	Cerebellum	VER10	Lobule X of vermis
16	Sensorimotor	lPreCG	Left Precentral gyrus
16	Sensorimotor	rPreCG	Right Precentral gyrus
16	Sensorimotor	lSMA	Left Supplementary motor area
16	Sensorimotor	rSMA	Right Supplementary motor area
16	Sensorimotor	lPoCG	Left Postcentral gyrus
16	Sensorimotor	rPoCG	Right Postcentral gyrus
17	Broca'area	lIFGoperc	Left Inferior frontal gyrus-opercular part
17	Broca'area	rIFGoperc	Right Inferior frontal gyrus-opercular part
17	Broca'area	lIFGtriang	Left Inferior frontal gyrus-triangular part
17	Broca'area	rIFGtriang	Right Inferior frontal gyrus-triangular part

Supplementary Table 8. Description of SuStaIn features.

Biomarker	Count	Mean	STD	Rank 50%	Rank 75%	Max
Hippocampus	4222	0.498	1.140	0.477	1.200	4.869
Parahippocampus	4222	0.347	1.067	0.336	1.053	4.439
Amygdala	4222	0.355	1.107	0.337	1.051	4.896
Caudate	4222	0.031	1.110	0.061	0.756	4.732
Putamen	4222	-0.137	1.111	-0.141	0.596	3.960
Pallidum	4222	-0.488	1.206	-0.343	0.335	2.797
Thalamus	4222	0.282	1.142	0.228	1.013	4.599
Accumbens	4222	0.152	1.105	0.165	0.894	4.224
Cingulate	4222	0.396	1.100	0.412	1.088	4.811
Frontal Cortex	4222	0.456	1.142	0.468	1.176	4.945
Parietal Cortex	4222	0.292	1.099	0.289	1.036	4.625
Temporal Cortex	4222	0.502	1.180	0.465	1.266	4.922
Occipital Cortex	4222	0.339	1.096	0.349	1.057	4.105
Insula	4222	0.478	1.128	0.470	1.233	4.665
Cerebellum	4222	0.180	1.048	0.168	0.872	4.743
Sensorimotor	4222	0.350	1.109	0.357	1.080	4.587
Broca'area	4222	0.368	1.013	0.380	1.046	4.529

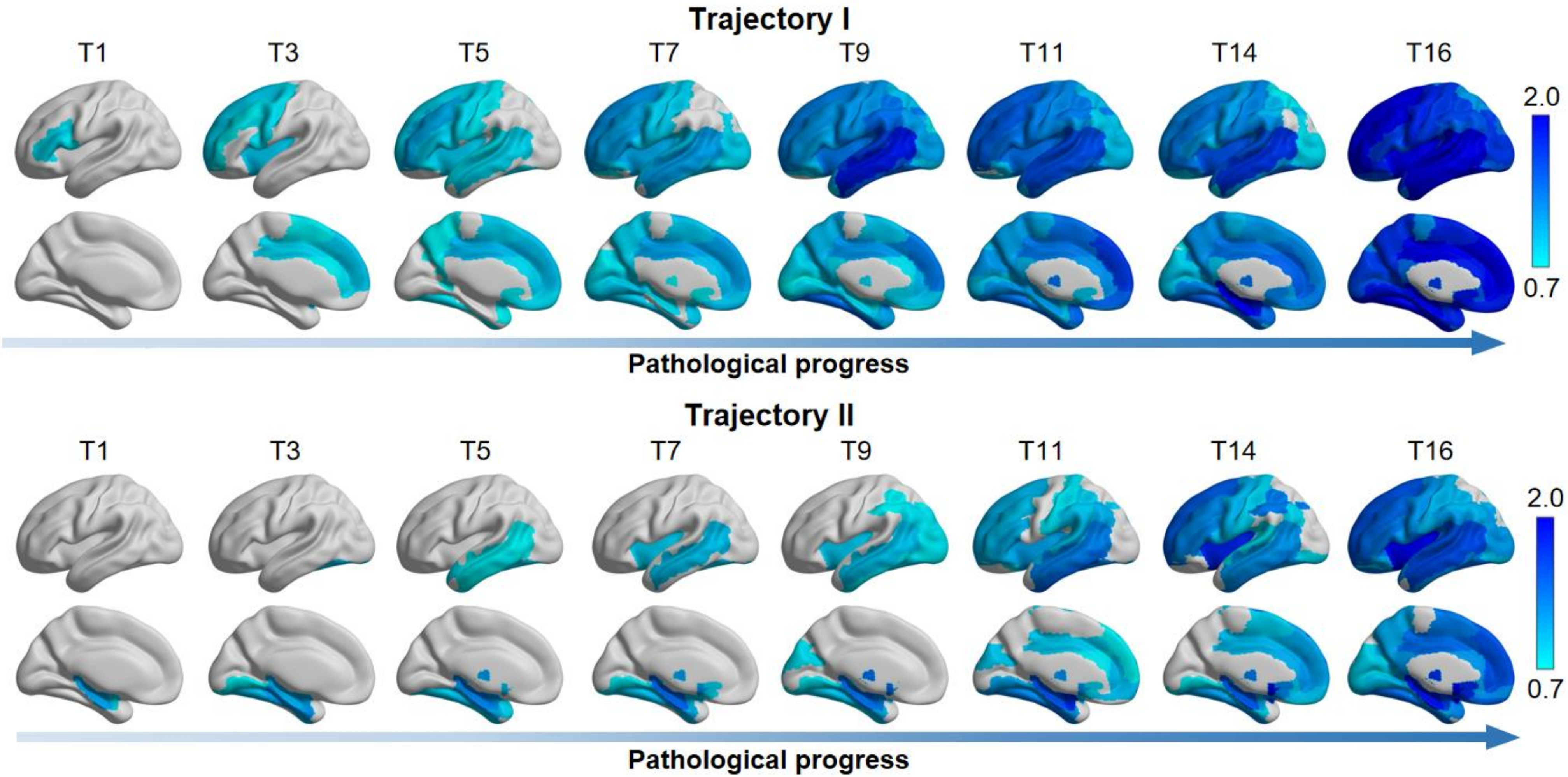

Supplementary Figure 1. Pathophysiological progression trajectories in validation dataset. Trajectories are repeated based on the additional dataset that has removed original data used in a previous SuStaIn study.

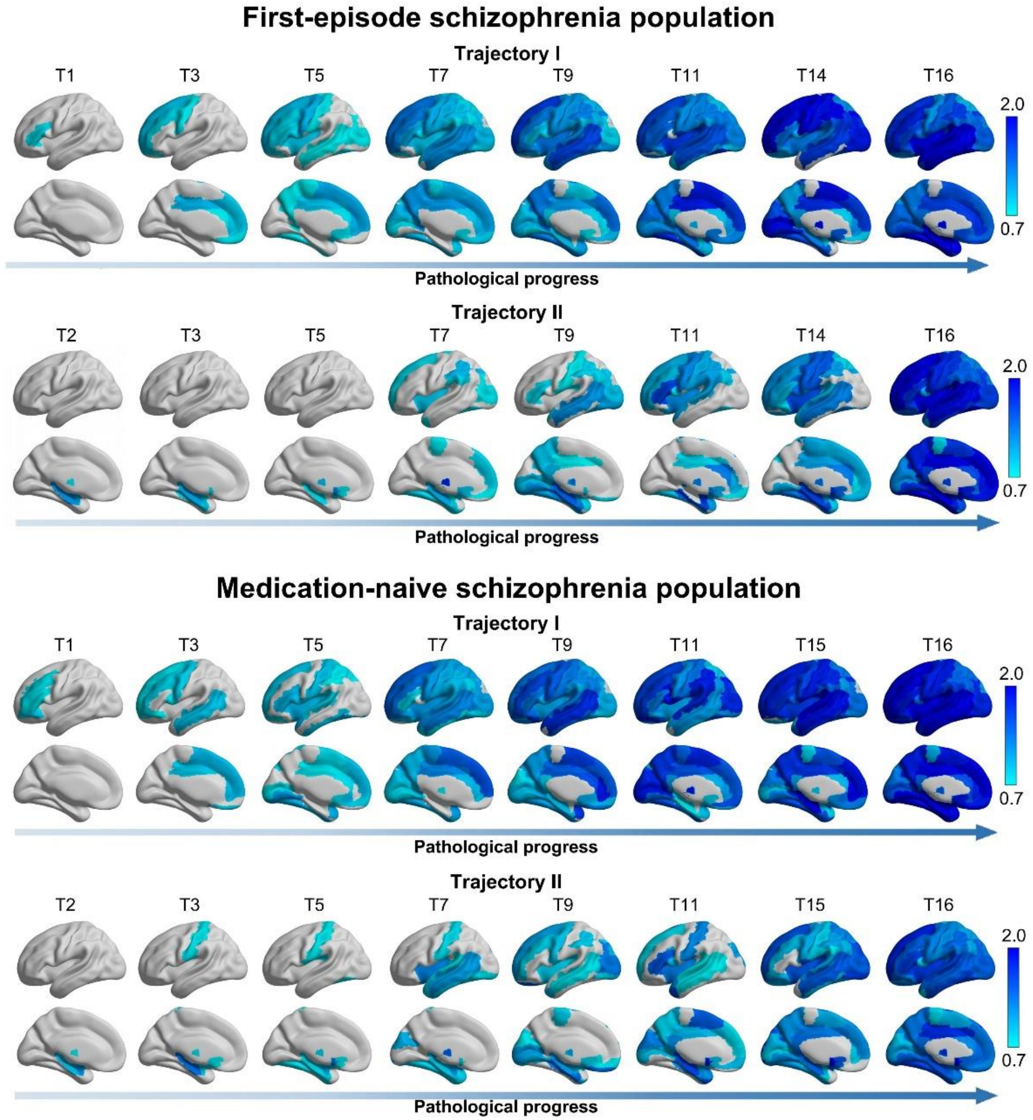

Supplementary Figure 2. Pathophysiological progression trajectories in first-episode population and medication-naïve population. Trajectories are repeated based on the subsample data from the first-episode schizophrenia patients whose illness duration was less than two years (*N*=1,112, 513 females, mean age=25.4± 12.4 years), and another subsample data from medication-naïve patients with schizophrenia (*N*=718, 353 females, mean age=23.7±12.1 years).

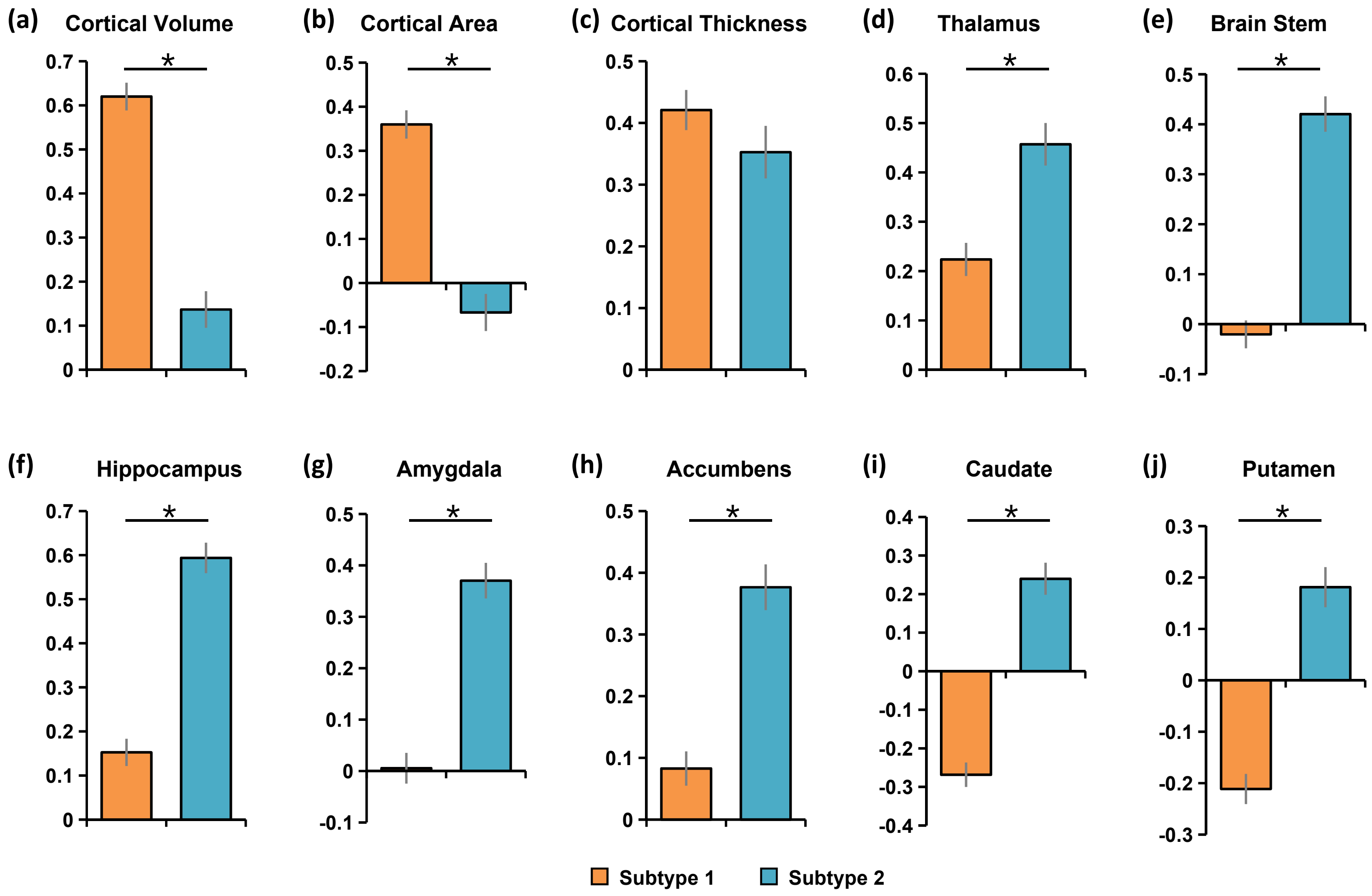

Supplementary Figure 3. Comparisons of morphological z-score between the two subtypes. A larger positive z-score indicates a larger deviation of reduction relative to healthy control group. Two sample t test is conducted to examine inter-subtype difference for the (**a**) averaged cortical volume (t=9.36, p<10e-16, Cohen's d=0.446); (**b**) averaged cortical area (t=8.09, p<10e-16, Cohen's d=0.386); (**c**) averaged cortical thickness (t=1.29, p=0.198, Cohen's d=0.061); (**d**) thalamus volume (t=-4.28, p=1.97e-5, Cohen's d=-0.205); (**e**) brain stem volume (t=-9.79, p<10e-16, Cohen's d=-0.469); (**f**) hippocampus volume (t=-9.25, p<10e-16, Cohen's d=-0.449); (**g**) amygdala volume (t=-7.83, p=8.44e-15, Cohen's d=-0.379); (**h**) accumbens volume (t=-6.40, p=1.94e-10, Cohen's d=-0.305); (**i**) caudate volume (t=-9.82, p<10e-16, Cohen's d=-0.468); (**j**) putamen volume (t=-8.14, p<10e-16, Cohen's d=-0.389). * p<0.05, two-sided, family wise error (FWE) correction.

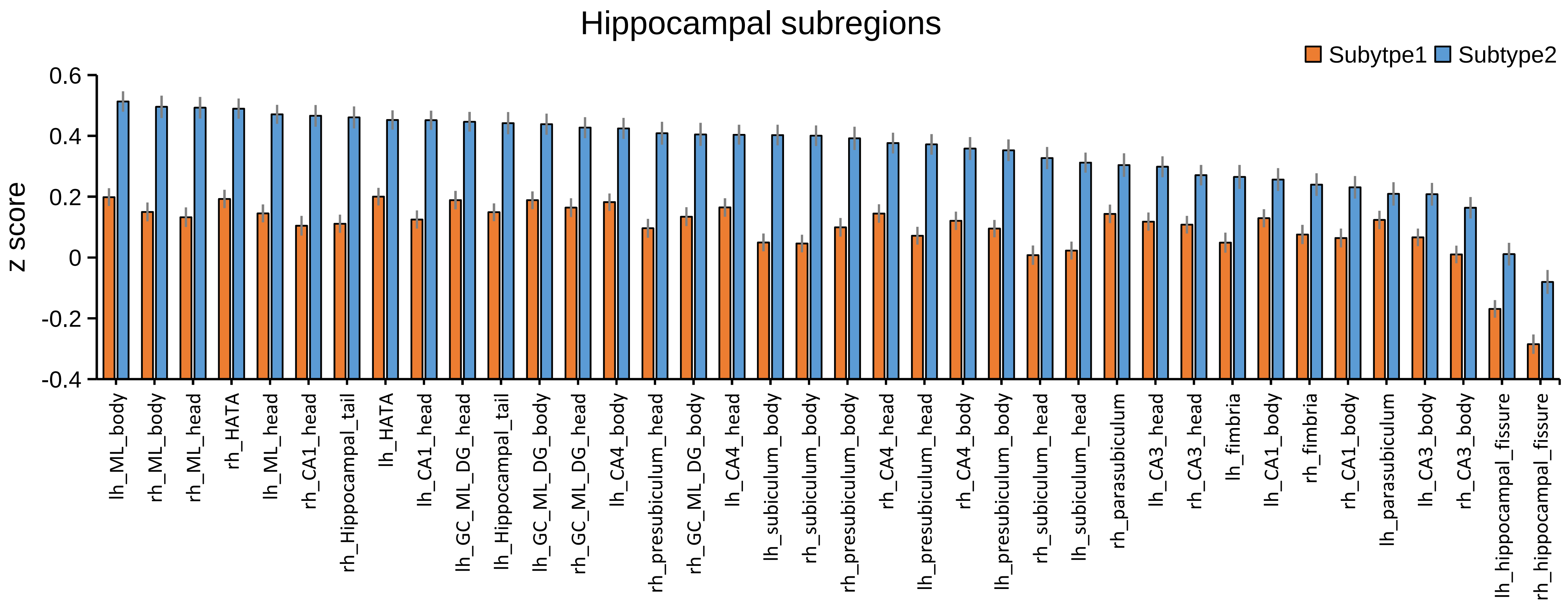

Supplementary Figure 4. Hippocampus subregional morphological z-score for the two subtypes. A larger positive z-score indicates a larger deviation of reduction relative to healthy control group.

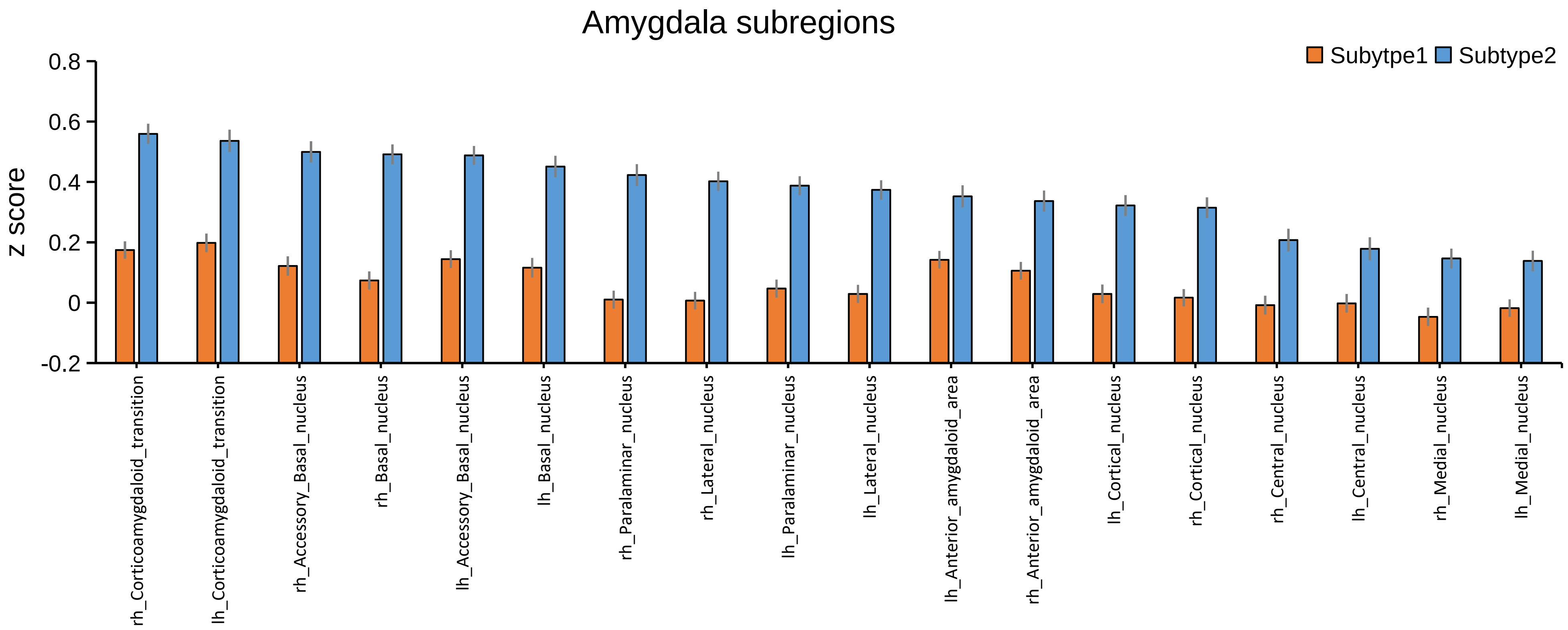

Supplementary Figure 5. Amygdala subregional morphological z-score for the two subtypes. A larger positive z-score indicates a larger deviation of reduction relative to healthy control group.

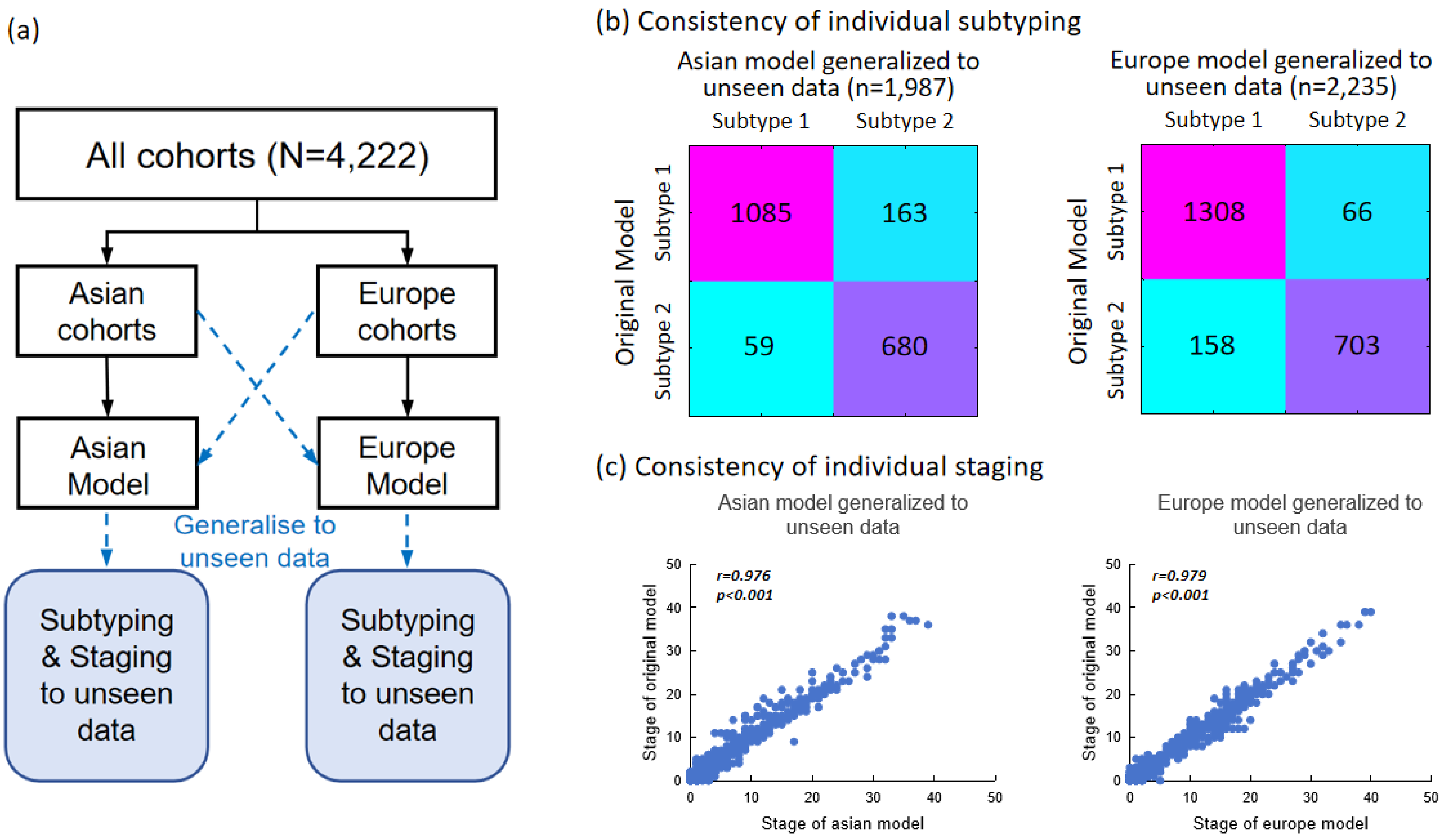

Supplementary Figure 6. Generalization of SuStaIn subtyping and staging to unseen cohorts. (**a**) The Asian and Europe SuStaIn models are separately built based on the Asian ancestry cohorts and Europe ancestry cohorts. The two models are used to subtyping and staging those unseen samples. We compare whether those subtype and stage assignments match the result of original model that is built on all cohorts. (**b**) Most of unseen individuals keep the same subtype label with the original model (88.83% for Asian model; 89.98% for Europe model). (**c**) A high consistency of individual staging between stages of unseen data and original model result (Asian model, r=0.976, $p<10^{-300}$; Europe model, r=0.979, $p<10^{-300}$, Spearman correlation test, two sided).

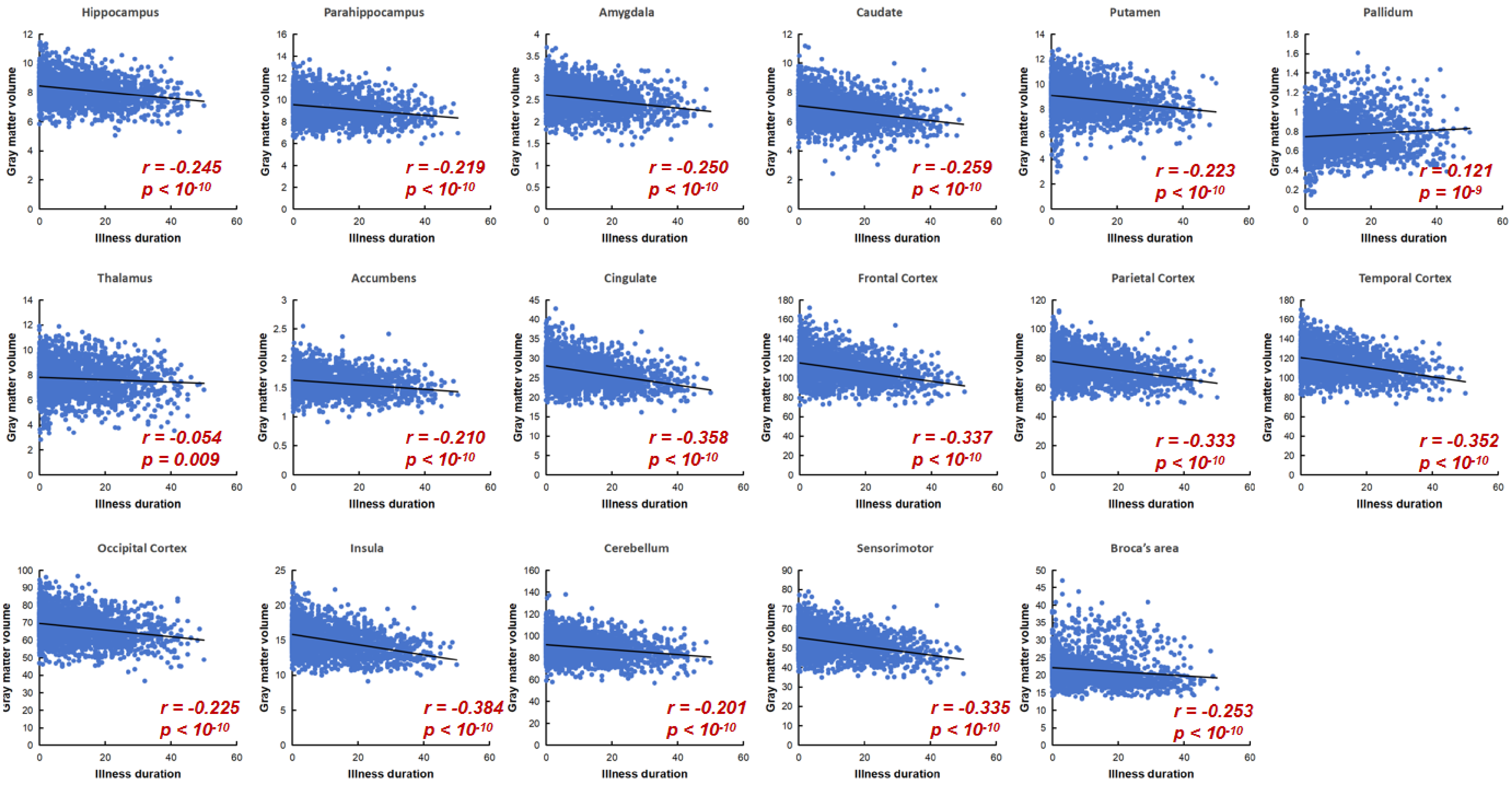

Supplementary Figure 7. Significant association between regional gray matter volume change and illness duration in 2,333 patients with schizophrenia.

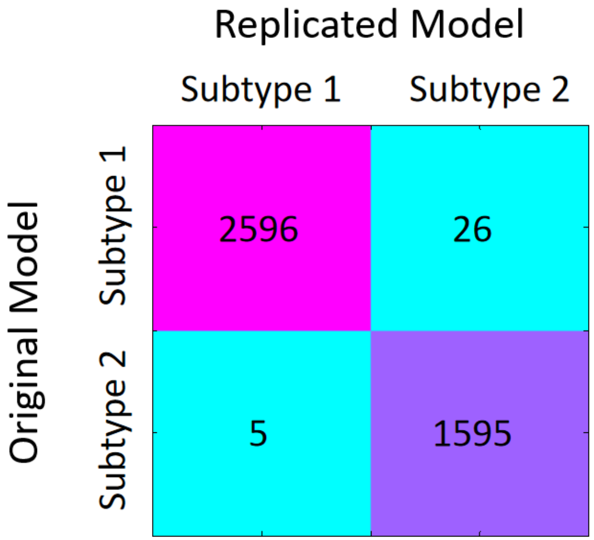

Supplementary Figure 8. The consistency of individual classification in both original model and replicated model. The maximum z-score in the SuStaIn algorithm is defined at *z*=5 and *z*=4 separately for original model and replicated model. A total of 4,191 individuals (99.27%) are assigned to the same subtype label.

Acknowledgements per dataset

Acknowledgements per dataset are as follows:

IGP: Supported by NHMRC Project Grants (630471 & 1081603).

ESO: Supported by Ministry of Health of the Czech Republic, grants nr. NU21-08-00432 and NU20-04-00393. Supported by Ministry of Health, Czech Republic - DRO 2021 ("Institute for Clinical and Experimental Medicine - IKEM, IN: 00023001").

FOR2107: Funded by the German Research Foundation (Deutsche Forschungsgemeinschaft DFG; Forschungsgruppe/Research Unit FOR2107). Principal investigators (PIs) with respective areas of responsibility in the FOR2107 consortium are: Work Package WP1, FOR2107/MACS cohort and brainimaging: Tilo Kircher (speaker FOR2107; DFG grant numbers KI588/14-1, KI588/14-2, KI588/20-1, KI588/22-1), Udo Dannlowski (co-speaker FOR2107; DA 1151/5-1, DA 1151/5-2, DA1151/6-1), Axel Krug (KR 3822/5-1, KR 3822/7-2), Igor Nenadic (NE2254/1-2, NE2254/3-1, NE2254/4-1, NE2254/2-1), Carsten Konrad (KO 4291/3-1).

IMH: Supported by research grants from the National Healthcare Group, Singapore (SIG/05004; SIG/05028), and the Singapore Bioimaging Consortium (RP C009/2006).

JBUN: Supported by Korean Mental Health Technology R&D Project, Ministry of Health & Welfare, Republic of Korea (HL19C0015) and a grant of the Korea Health Technology R&D Project through the Korea Health Industry Development Institute (KHIDI), funded by the Ministry of Health & Welfare, Republic of Korea (HR18C0016).

OLIN: Supported by MH106324.

Osaka: Supported by JSPS KAKENHI (Grant Number JP21K12153), ABiS (JSPS KAKENHI Grant Number JP22H04926), Brain/MINDS & beyond studies (Grant Number JP18dm0307002) from the AMED.

PENS: Supported by department of Veterans Affairs CSR&D (I01CX000227) and National Institutes of Health (U01MH108150) to Scott R. Sponheim. RomeSL: Supported by Italian Ministry of Health, grant Ricerca Corrente RC 23, RF-2019-12370182.

SoCAT: Supported by THE SCIENTIFIC AND TECHNOLOGICAL RESEARCH COUNCIL OF TÜRKİYE.

SWIFT: Supported by a NARSAD grant from the Brain & Behavior Research Foundation (28445) and by a Research Grant from the Novartis Foundation (20A058).

UCISZ: Supported by the National Institute of Mental Health of the National Institutes of Health under award number R21MH097196.

UNINA: Supported by #NEXTGENERATIONEU (NGEU) and funded by the Ministry of University and Research (MUR), National Recovery and Resilience Plan (NRRP), project MNESYS (PE0000006) – A Multiscale integrated approach to the study of the nervous system in health and disease (DN. 1553 11.10.2022).

COBRE: Supported by NIMH: 5R01MH094524-1; 3R01MH121246; 1P20RR021938.

TOPSY: Supported by the Canada First Research Excellence Fund, awarded to the Healthy Brains, Healthy Lives initiative at McGill University (through a New Investigator Supplement) and the Monique H. Bourgeois Chair in Developmental Disorders. He receives a salary award from the Fonds de recherche du Québec - Santé (FRQS).

OSLO: Supported by The Research Council of Norway (249795, 300767), the South-Eastern Norway Regional Health Authority (2014097, 2019101), KG Jebsen Stiftelsen, the European Research Council under the European Union s Horizon 2020 research and Innovation program (802998), and the European Union-funded Horizon Europe project ‘environMENTAL’ (no. 101057429).

HCP-EP: Research using Human Connectome Project for Early Psychosis (HCP-EP) data reported in this publication was supported by the National Institute of Mental Health of the National Institutes of Health under Award Number U01MH109977.

JP-SRPBS: Data used in the preparation of this work were obtained from the DecNef Project Brain Data Repository (https://bicr-resource.atr.jp/srpbsopen/) gathered by a consortium as part of the Japanese Strategic Research Program for the Promotion of Brain Science (SRPBS) supported by the Japanese Advanced Research and Development Programs for Medical Innovation (AMED).

FBIRN: Data used for this study were downloaded from the FunctionBIRN Data Repository, supported by grants to the Function BIRN (U24-RR021992) Testbed funded by theNational Center for Research Resources at the National Institutes of Health, U.S.A.

MCIC: The imaging data and demographic information was collected and shared by [University of Iowa, University of Minnesota, University of New Mexico, Massachusetts General Hospital] the Mind Research Network supported by the Department of Energy under Award Number DE-FG02-08ER64581.

NMorphCH: Data collection and sharing was funded by NIMH grant R01 MH056584.

NUSDAST: Data collection and sharing was funded by NIMH grant 1R01 MH084803.

References

1. Morgan, C., et al., *Rethinking the course of psychotic disorders: modelling long-term symptom trajectories.* Psychol Med, 2021: p. 1-10.
2. Austin, S.F., et al., *Long-term trajectories of positive and negative symptoms in first episode psychosis: A 10year follow-up study in the OPUS cohort.* Schizophr Res, 2015. **168**(1-2): p. 84-91.
3. Dukart, J., et al., *Age-related brain structural alterations as an intermediate phenotype of psychosis.* J Psychiatry Neurosci, 2017. **42**(5): p. 307-319.
4. Guo, S., et al., *Dynamic cerebral reorganization in the pathophysiology of schizophrenia: a MRI-derived cortical thickness study.* Psychol Med, 2016. **46**(10): p. 2201-14.
5. Lv, J., et al., *Individual deviations from normative models of brain structure in a large cross-sectional schizophrenia cohort.* Mol Psychiatry, 2021. **26**(7): p. 3512-3523.
6. Lepage, M., et al., *Do Unremitted Psychotic Symptoms Have an Effect on the Brain? A 2-Year Follow-up Imaging Study in First-Episode Psychosis.* Schizophr Bull Open, 2020. **1**(1): p. sgaa039.
7. Andreasen, N.C., et al., *Relapse duration, treatment intensity, and brain tissue loss in schizophrenia: a prospective longitudinal MRI study.* Am J Psychiatry, 2013. **170**(6): p. 609-15.
8. Schennach, R., et al., *What are residual symptoms in schizophrenia spectrum disorder? Clinical description and 1-year persistence within a naturalistic trial.* European Archives of Psychiatry and Clinical Neuroscience, 2015. **265**(2): p. 107-116.
9. Koutsouleris, N., et al., *Exploring Links Between Psychosis and Frontotemporal Dementia Using Multimodal Machine Learning: Dementia Praecox Revisited.* JAMA Psychiatry, 2022.
10. Bethlehem, R.A.I., et al., *Brain charts for the human lifespan.* Nature, 2022. **604**(7906): p. 525-533.
