# Supplementary material for: Neurostructural subgroup in 4291 individuals with schizophrenia identified using the subtype and stage inference algorithm
Source: Nat Commun. 2024 Jul 17;15:5996. doi: 10.1038/s41467-024-50267-3 (PMC11252381; doi:10.1038/s41467-024-50267-3)
Supplement: Supplementary file 3 — Description of Additional Supplementary Files [file 41467_2024_50267_MOESM3_ESM.pdf]

Description of Additional Supplementary Files

File Name: Supplementary Movie 1
Description: The progressive pattern of spatial expansion along with later ‘temporal’ stages of pathological progression for the ‘trajectory’ 1, which displays an ‘early cortical-predominant loss’ biotype.

File Name: Supplementary Movie 2
Description: The progressive pattern of spatial expansion along with later ‘temporal’ stages of pathological progression for the ‘trajectory’ 2, which displays an ‘early subcortical-predominant loss’ biotype.
