# Supplementary figures and images for: Neurostructural subgroup in 4291 individuals with schizophrenia identified using the subtype and stage inference algorithm
Source: Nat Commun. 2024 Jul 17;15:5996. doi: 10.1038/s41467-024-50267-3 (PMC11252381; doi:10.1038/s41467-024-50267-3)

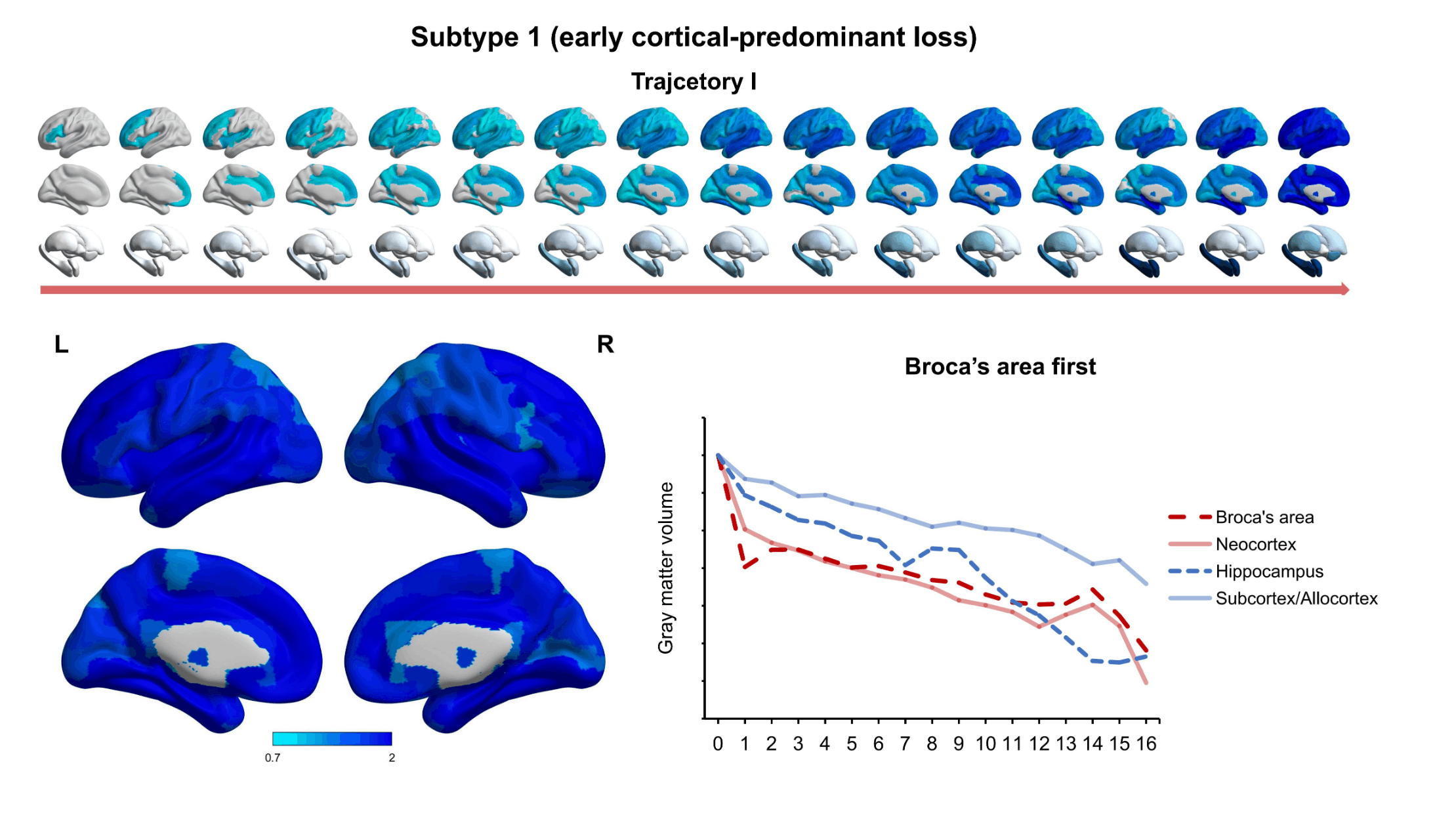

Supplement: Supplementary file 4 — Supplementary Movie 1 [file 41467_2024_50267_MOESM4_ESM.gif]

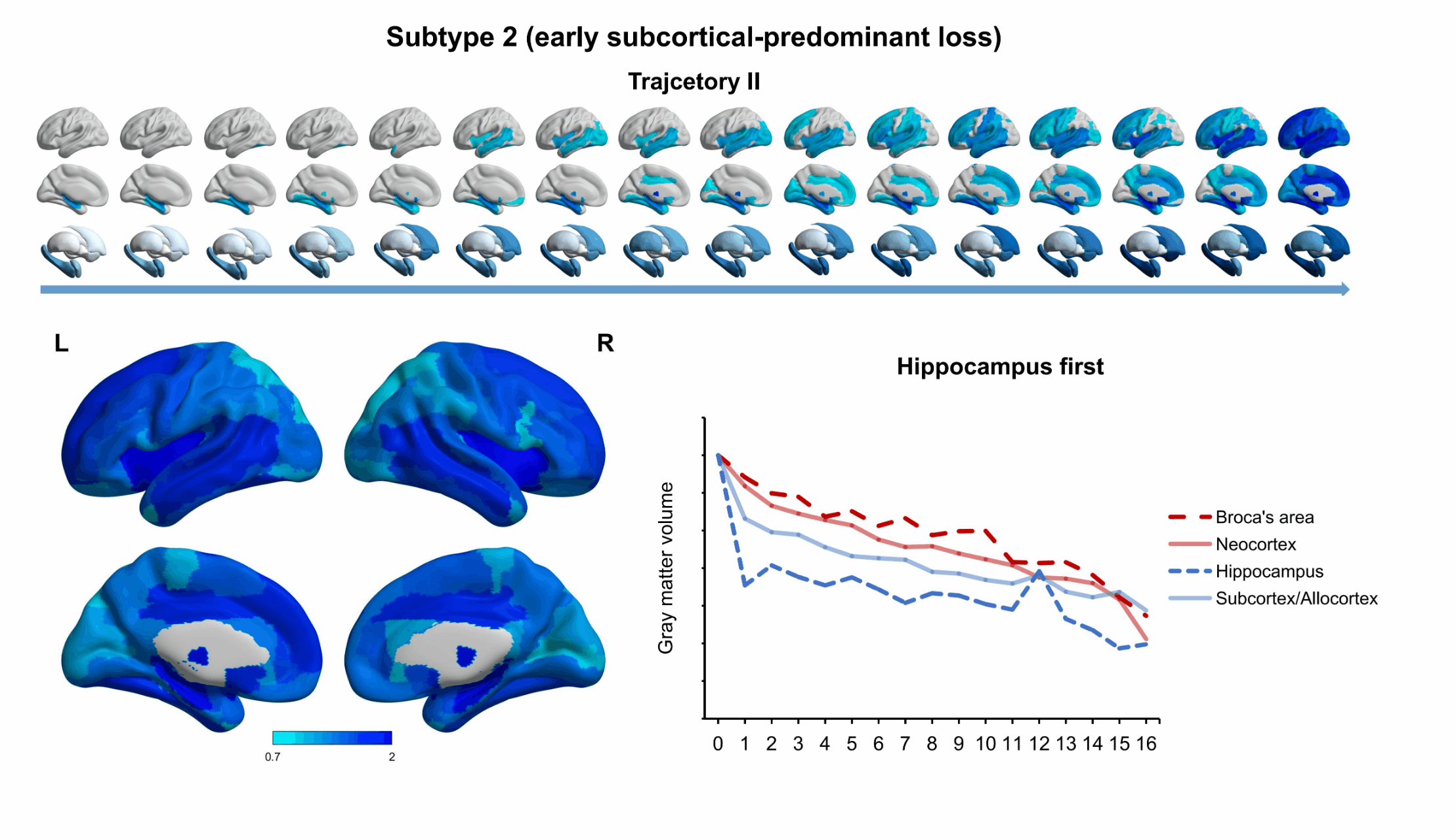

Supplement: Supplementary file 5 — Supplementary Movie 2 [file 41467_2024_50267_MOESM5_ESM.gif]
