# Supplementary material for: Neurostructural subgroup in 4291 individuals with schizophrenia identified using the subtype and stage inference algorithm
Source: Nat Commun. 2024 Jul 17;15:5996. doi: 10.1038/s41467-024-50267-3 (PMC11252381; doi:10.1038/s41467-024-50267-3)
Supplement: Supplementary file 6 — Reporting Summary [file 41467_2024_50267_MOESM6_ESM.pdf]

nature portfolio

Corresponding author(s): Jianfeng Feng

Last updated by author(s): May 27, 2024

Reporting Summary

Nature Portfolio wishes to improve the reproducibility of the work that we publish. This form provides structure for consistency and transparency in reporting. For further information on Nature Portfolio policies, see our Editorial Policies and the Editorial Policy Checklist.

Statistics

For all statistical analyses, confirm that the following items are present in the figure legend, table legend, main text, or Methods section.

n/a	Confirmed	
☐	☒	The exact sample size (*n*) for each experimental group/condition, given as a discrete number and unit of measurement
☐	☒	A statement on whether measurements were taken from distinct samples or whether the same sample was measured repeatedly
☐	☒	The statistical test(s) used AND whether they are one- or two-sided<br>*Only common tests should be described solely by name; describe more complex techniques in the Methods section.*
☐	☒	A description of all covariates tested
☐	☒	A description of any assumptions or corrections, such as tests of normality and adjustment for multiple comparisons
☐	☒	A full description of the statistical parameters including central tendency (e.g. means) or other basic estimates (e.g. regression coefficient) AND variation (e.g. standard deviation) or associated estimates of uncertainty (e.g. confidence intervals)
☐	☒	For null hypothesis testing, the test statistic (e.g. *F*, *t*, *r*) with confidence intervals, effect sizes, degrees of freedom and *P* value noted<br>*Give P values as exact values whenever suitable.*
☐	☒	For Bayesian analysis, information on the choice of priors and Markov chain Monte Carlo settings
☒	☐	For hierarchical and complex designs, identification of the appropriate level for tests and full reporting of outcomes
☐	☒	Estimates of effect sizes (e.g. Cohen's *d*, Pearson's *r*), indicating how they were calculated

Our web collection on statistics for biologists contains articles on many of the points above.

Software and code

Policy information about availability of computer code

Data collection	This study analyzed cross-sectional T1-weighted structural MRI data from a total of 4,291 individuals diagnosed with schizophrenia (1,709 females, mean age=32.5±11.9 years) and 7,078 healthy controls (3,461 females, mean age=33.0±12.7 years). These datasets came from 21 cohorts of ENIGMA schizophrenia working groups from various countries around the world, 11 cohorts collected from Chinese hospitals over the last ~10 years, and 9 cohorts from publicly available datasets, i.e., HCP-EP , JP-SRPBS , fBIRN, MCIC, NMorphCH , NUSDAST, DS000030 , DS000115 and DS004302.
Data analysis	T1-weighted structural brain MRI scans were acquired at each study site. We used a standardized protocol for image processing using the ENIGMA Computational Anatomy Toolbox (CAT12) across multiple cohorts (https://neuro-jena.github.io/enigma-cat12/). These protocols enable region-based gray matter volume (GMV) measures for image data based on the automated anatomical (AAL3) atlas.

For manuscripts utilizing custom algorithms or software that are central to the research but not yet described in published literature, software must be made available to editors and reviewers. We strongly encourage code deposition in a community repository (e.g. GitHub). See the Nature Portfolio guidelines for submitting code & software for further information.

Data

Policy information about availability of data

All manuscripts must include a data availability statement. This statement should provide the following information, where applicable:

- Accession codes, unique identifiers, or web links for publicly available datasets
- A description of any restrictions on data availability
- For clinical datasets or third party data, please ensure that the statement adheres to our policy

The raw image and clinical data are protected and are not available due to data privacy laws. The processed data are available through the following links. Data of NMorphCH, FBIRN and NUSDAST were obtained from the SchizConnect, a publicly available website (http://www.schizconnect.org/documentation#by_project). The NMorphCH dataset and NUSDAST dataset were download through a query interface at the SchizConnect (http://www.schizconnect.org/queries/new). The FBIRN dataset was download from https://www.nitrc.org/projects/fbirn/. The DS000115 dataset was download from OpenfMRI database (https://www.openfmri.org/). The DS000030 dataset was available at https://legacy.openfmri.org/dataset/ds000030/. The DS004302 dataset was available at https://openneuro.org/datasets/ds004302/versions/1.0.1. The HCP-EP dataset was available at https://www.humanconnectome.org/study/human-connectome-project-for-early-psychosis/. The Japanese SRPBS Multi-disorder MRI Dataset was available at https://bicr-resource.atr.jp/srpbsopen/. Requests for ENIGMA data can be applied via the ENIGMA Schizophrenia Working Group (https://enigma.ini.usc.edu/ongoing/enigma-schizophrenia-working-group/). The statistical data generated in this study are provided in the Supplementary Information/Source Data file.

Research involving human participants, their data, or biological material

Policy information about studies with human participants or human data. See also policy information about sex, gender (identity/presentation), and sexual orientation and race, ethnicity and racism.

Reporting on sex and gender	Term 'sex' was used due to the biological attribution. The sex was determined based on self-reporting. This study analyzed cross-sectional T1-weighted structural MRI data from a total of 4,291 individuals diagnosed with schizophrenia (1,709 females, mean age=32.5±11.9 years) and 7,078 healthy controls (3,461 females, mean age=33.0±12.7 years).
Reporting on race, ethnicity, or other socially relevant groupings	As samples came from different parts of the world, we divided all samples into several sub-cohorts based on where the samples were obtained. Samples from China, Japan, South Korea and Singapore were classified into the East Asian ancestry (EAS) cohort. Samples from Europe, the United States, Canada and Australia were classified into the European ancestry (EUR) cohorts (Supplementary Table 4).
Population characteristics	This information is provided in detail in Supplementary Table 1-2 and Supplementary Materials, but for a summary: cross-sectional T1-weighted structural MRI data came from a total of 4,291 individuals diagnosed with schizophrenia (1,709 females, mean age=32.5±11.9 years) and 7,078 healthy controls (3,461 females, mean age=33.0±12.7 years).
Recruitment	Details of demographics, geographic location, clinical characteristics, and inclusion/exclusion criteria for each cohort may be found in the Supplementary Information (Supplementary Table S1-2).All sites obtained approval from their local institutional review boards or ethics committees, and written informed consent from all participants and/or their legal guardians.
Ethics oversight	The present study was carried out under the approve from the Medical Research Ethics Committees of Fudan University (Number: FE222711).

Note that full information on the approval of the study protocol must also be provided in the manuscript.

Field-specific reporting

Please select the one below that is the best fit for your research. If you are not sure, read the appropriate sections before making your selection.

☒ Life sciences ☐ Behavioural & social sciences ☐ Ecological, evolutionary & environmental sciences

For a reference copy of the document with all sections, see nature.com/documents/nr-reporting-summary-flat.pdf

Life sciences study design

All studies must disclose on these points even when the disclosure is negative.

Sample size	Because comprehensive effect size/power calculations have not been comprehensively investigated for SuStaIn, the sample size was determined according to previous SuStaIn studies (Young et al., 2018, Nature Communications and Vogel et al., 2021, Nature Medicine).As these previous studies indicated that about 30-40% subjects would actually exhibit measurable abnormal gray matter volume reductions. For this reason, it was important for us to analyze data from at least 600 patients with schizophrenia, and ideally greater than 1000. The sample size for this study is over 11,000 participants (over 4,000 patients with schizophrenia), which gives us broad representation across the disease progress for SuStaIn modeling.
Data exclusions	Individuals were excluded from the study if they were (1) diagnosed with schizoaffective disorder, mood disorders, or other major medical or neurologic disorders; (2) alcohol/drug dependence; (3) had a history of electroconvulsive therapy within six months; (4) other contraindications to MRI scanning.<br>As the samples included multi-center data with possibly large variation, it is important to conduct a protocol to ensure data quality. The data quality control (QC) steps are described as follows: (1) QC for raw T1-weighted images. Raw T1-weighted structural brain images were

	checked for large distortions, ghosting or other abnormalities by at least two experienced neuroradiologists. (2) QC for image processing. Raw T1-weighted structural brain images were processed with the Computational Anatomy Toolbox (CAT) within SPM (http://www.neuro.uni-jena.de/cat/). The results of segment and spatial normalization were visually inspected by at least two experienced specialists in brain image processing. (3) After data processing, CAT reported a weighted average image quality rating to exclude those subjects with poor quality. These quality control steps allowed us to exclude those subjects with poor quality and generate a subject list for the following analysis. For data from enigma, we have no access to raw images . We removed these samples if they were marked as a statistical outlier (>5 standard deviations away from the global mean). After the quality control, 11,260 individuals were included, of which 4,222 were schizophrenia patients (1,683 females, mean age=32.4±12.4 years) and 7,038 healthy subjects (3,440 females, mean age=33.0±12.4 years).
Replication	To evaluate the consistency of the SuStaIn model, we used 2-fold cross-validation method. The cohort was randomly split into two non-overlapping subfolds (50% of the subjects as one subfold and left 50% as the other subfold). This procedure was repeated ten times to avoid the occasionality of one split. For each non-overlapping subfold, the SuStaIn model was trained on one of non-overlapping folds, and further tested using the other non-overlapping subfold.
Randomization	This is a retrospective study based on existing data; therefore randomization is not relevant in this study.
Blinding	Blinding was not relevant for experiments because we used a data-driven method.

Reporting for specific materials, systems and methods

We require information from authors about some types of materials, experimental systems and methods used in many studies. Here, indicate whether each material, system or method listed is relevant to your study. If you are not sure if a list item applies to your research, read the appropriate section before selecting a response.

Materials & experimental systems

n/a	Involved in the study	
☒	☐	Antibodies
☒	☐	Eukaryotic cell lines
☒	☐	Palaeontology and archaeology
☒	☐	Animals and other organisms
☒	☐	Clinical data
☒	☐	Dual use research of concern
☒	☐	Plants

Methods

n/a	Involved in the study	
☒	☐	ChIP-seq
☒	☐	Flow cytometry
☐	☒	MRI-based neuroimaging

Plants

Seed stocks	N/A
Novel plant genotypes	N/A
Authentication	N/A

Magnetic resonance imaging

Experimental design

Design type	This is not relevant because subjects did not need to perform experiment.
Design specifications	This is not relevant because subjects did not need to perform experiment.
Behavioral performance measures	This is not relevant because subjects did not need to perform experiment.

Acquisition

Imaging type(s)	structural
Field strength	3T;1.5T;7T
Sequence & imaging parameters	Details of image acquisition parameters are described in Supplementary Table S2.
Area of acquisition	whole brain scan

Diffusion MRI ☐ Used ☒ Not used

Preprocessing

Preprocessing software	T1-weighted structural brain MRI scans were acquired at each study site. We used a standardized protocol for image processing using the ENIGMA Computational Anatomy Toolbox (CAT12) across multiple cohorts (https://neuro-jena.github.io/enigma-cat12/).
Normalization	Preprocessing steps including spatial registration, tissue segmentation and bias correction of intensity non-uniformities were conducted using CAT12.
Normalization template	Preprocessing steps including spatial registration, tissue segmentation and bias correction of intensity non-uniformities were conducted using CAT12.
Noise and artifact removal	Preprocessing steps including spatial registration, tissue segmentation and bias correction of intensity non-uniformities were conducted using CAT12.
Volume censoring	Preprocessing steps including spatial registration, tissue segmentation and bias correction of intensity non-uniformities were conducted using CAT12.

Statistical modeling & inference

Model type and settings	A novel data-driven approach – Subtype and Stage Inference (SuStaIn) was used to perform disease progression modeling and cluster individuals while accounting for disease progression. The SuStaIn has been described previously (Young et al 2018 Nature Communications).
Effect(s) tested	This is not relevant because this study is a structural MRI study rather than task fMRI.

Specify type of analysis: ☐ Whole brain ☒ ROI-based ☐ Both

Statistic type for inference (See Eklund et al. 2016)	This is not relevant because GMV value was extracted at ROI-level.
Correction	FWE

Models & analysis

n/a	Involved in the study
☒	☐ Functional and/or effective connectivity
☒	☐ Graph analysis
☒	☐ Multivariate modeling or predictive analysis

Functional and/or effective connectivity	*Report the measures of dependence used and the model details (e.g. Pearson correlation, partial correlation, mutual information).*
Graph analysis	*Report the dependent variable and connectivity measure, specifying weighted graph or binarized graph, subject- or group-level, and the global and/or node summaries used (e.g. clustering coefficient, efficiency, etc.).*
Multivariate modeling and predictive analysis	*Specify independent variables, features extraction and dimension reduction, model, training and evaluation metrics.*
